# Antibiotic-producing symbionts dynamically transition between plant pathogenicity and insect-defensive mutualism

**DOI:** 10.1038/ncomms15172

**Published:** 2017-04-28

**Authors:** Laura V. Flórez, Kirstin Scherlach, Paul Gaube, Claudia Ross, Elisabeth Sitte, Cornelia Hermes, Andre Rodrigues, Christian Hertweck, Martin Kaltenpoth

**Affiliations:** 1 Insect Symbiosis Research Group, Max Planck Institute for Chemical Ecology, Hans-Knöll-Straβe 8, 07745 Jena, Germany; 2 Department for Evolutionary Ecology, Institute of Organismic and Molecular Evolution, Johannes Gutenberg University, Johann-Joachim-Becher-Weg 13, 55128 Mainz, Germany; 3 Department of Biomolecular Chemistry, Leibniz Institute for Natural Products Research and Infection Biology, HKI, Beutenbergstraβe 11a, 07745 Jena, Germany; 4 Department of Biochemistry and Microbiology, UNESP-São Paulo State University, Av. 24A, n. 1515-Bela Vista, Rio Claro, São Paulo 13506-900, Brazil; 5 Chair for Natural Product Chemistry, Friedrich Schiller University, 07743 Jena, Germany

## Abstract

Pathogenic and mutualistic bacteria associated with eukaryotic hosts often lack distinctive genomic features, suggesting regular transitions between these lifestyles. Here we present evidence supporting a dynamic transition from plant pathogenicity to insect-defensive mutualism in symbiotic *Burkholderia gladioli* bacteria. In a group of herbivorous beetles, these symbionts protect the vulnerable egg stage against detrimental microbes. The production of a blend of antibiotics by *B. gladioli*, including toxoflavin, caryoynencin and two new antimicrobial compounds, the macrolide lagriene and the isothiocyanate sinapigladioside, likely mediate this defensive role. In addition to vertical transmission, these insect symbionts can be exchanged via the host plant and retain the ability to initiate systemic plant infection at the expense of the plant's fitness. Our findings provide a paradigm for the transition between pathogenic and mutualistic lifestyles and shed light on the evolution and chemical ecology of this defensive mutualism.

## Abstract (web summary)

Observations of recent or dynamic transitions between parasitism and mutualism are scarce. Here, Flórez *et al*. provide evidence that *Burkholderia gladioli* bacteria can protect the eggs of herbivorous beetles by producing antimicrobial compounds, while retaining their ancestral ability to infect plants.

Symbiosis is ubiquitous in nature and constitutes a major source of evolutionary innovation, playing a fundamental role in the origin and diversification of eukaryotic life on Earth^1^. Microbial symbionts influence virtually all aspects of eukaryote biology^2^, and their impact on host fitness ranges from detrimental to beneficial, occasionally shifting along this continuum^3^. Although such shifts have important implications for the ecological and evolutionary dynamics of symbiosis, observations of recent or dynamic transitions between parasitic and mutualistic lifestyles are scarce, and reports on their occurrence rely largely on phylogenetic evidence^4^. Furthermore, genomic analyses across bacterial groups reveal that general signatures distinguishing pathogenic and mutualistic microbes are often lacking^4^,^5^,^6^.

Here, we report on a transition between pathogenicity and mutualism in *Burkholderia* bacteria associated with a widespread group of herbivorous beetles, the Lagriinae (Coleoptera: Tenebrionidae). We show that these insect-defensive mutualists likely evolved from plant pathogenic bacteria, and that they can still infect a plant host, proposing an ecological context in which the lifestyle transition may have occurred. Furthermore, we elucidate symbiont-produced compounds that inhibit the growth of relevant microbial antagonists of the insect.

## Results

### *Burkholderia* as vertically transmitted symbionts in Lagriinae

**Figure 1:**
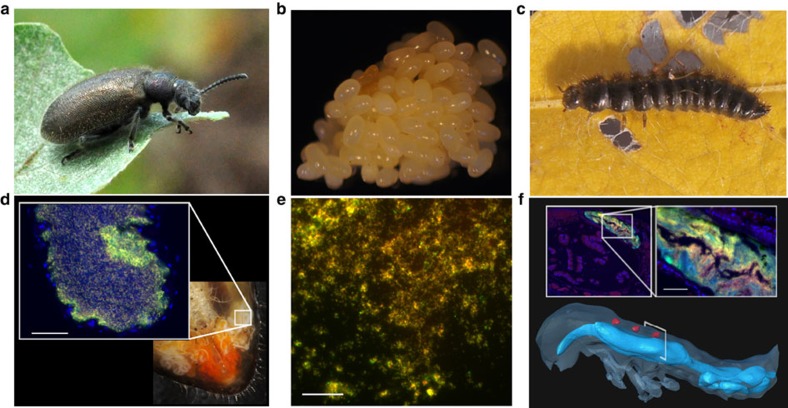
*Burkholderia gladioli* symbionts are transmitted vertically via egg smearing in *Lagria villosa*beetles. (**a**,**d**) Adult females carry the symbionts within two pairs of accessory glands associated with the reproductive system as confirmed by FISH on cross-sections of a female reproductive system (inset). (**b**,**e**) Host eggs are covered with a secretion containing *Burkholderia* bacteria, as revealed by FISH on an egg wash. (**c**,**f**) The symbionts colonize invaginations of the cuticle that result in three dorsal compartments represented in red in a three-dimensional (3D) reconstruction of an *L. hirta* larva. The *Burkholderia* symbionts were localized by FISH in a cross-section of an *L. villosa* larva (inset). FISH pictures show *Burkholderia*-specific staining in red (Burk16S_Cy3), general eubacterial staining in green (EUB338_Cy5), the overlap of these two in yellow and host cell nuclei in blue (DAPI). Scale bars, 20 μm (**d**) and 50 μm (**e**,**f**).

Lagriinae beetles harbour extracellular bacteria in a pair of accessory glands connected to the female reproductive system^7^ (Fig. 1a,d) that are transmitted vertically via the egg surface^7^ (Fig. 1b,e). Shortly before hatching, some bacterial cells enter the egg and colonize invaginations of the cuticle located dorsally in the embryo that later close to form three compartments in the larva^7^ (Fig. 1c,f). To identify the bacterial symbionts associated with the invasive South American soybean pest *Lagria villosa*^8^ and to confirm their vertical transmission route, we characterized the bacterial community in the symbiont-bearing structures of field-collected adult females and eggs laid by these females, as well as in larvae of laboratory cultures using 454 sequencing of bacterial 16S ribosomal RNA (rRNA) amplicons and quantitative PCR. We identified a closely related cluster of bacteria from the genus *Burkholderia* as the most prevalent taxon in female accessory glands and eggs (65–86% and 30–71% of reads per individual gland or egg clutch, respectively) (Supplementary Fig. 1a,b), and we found the same bacteria in mean abundances of 1.39 × 10^6^, 1.83 × 10^7^ and 1.59 × 10^8^ 16S rRNA gene copies per egg, larva and adult female gland, respectively (Supplementary Fig. 1c). Longer 16S rRNA reads (1.1–1.3 kb) obtained by Sanger sequencing revealed that the symbionts are most similar to *Burkholderia gladioli*, a well-known plant pathogen, and that at least three highly similar strains coinfect *L. villosa* beetles (Supplementary Fig. 2 and Supplementary Table 1). As supported by findings in the congeneric beetle species *Lagria hirta*, the coexistence of multiple symbiotic *B. gladioli* strains within individual beetles might be a common feature in Lagriinae beetles (Supplementary Fig. 2 and Supplementary Table 1). One of the strains could be successfully isolated from *L. villosa* and cultured *in vitro* (*B. gladioli* Lv-StA). Additionally, *Burkholderia*-specific fluorescence *in situ* hybridization (FISH) confirmed symbiont localization in the adult female reproductive glands, on the egg surface and in the unusual dorsal organs of larvae (Fig. 1).

### Egg antifungal protection by *B. gladioli* symbionts

**Figure 2:**
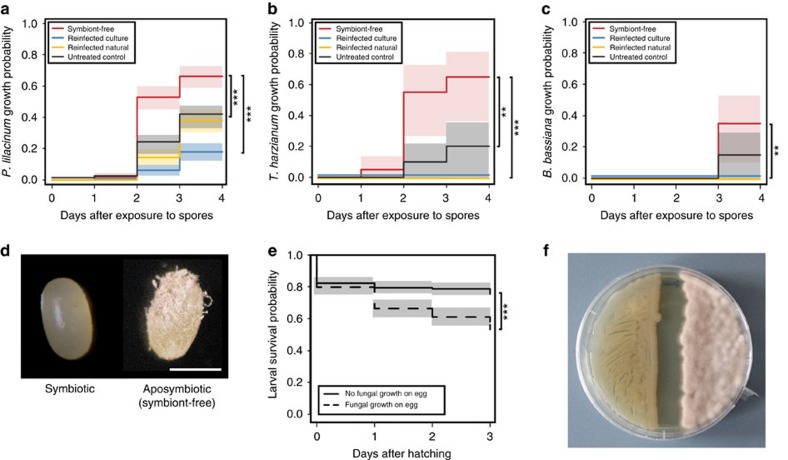
*B. gladioli* symbionts protect *L. villosa>* eggs from fungal infestation. In the absence of the symbionts on *L. villosa* eggs, there is a higher probability of the following three fungi to grow: (**a**) *Purpureocillium lilacinum* (*N*=180 per treatment, Cox mixed effects model, *P*<0.001 compared with all controls), (**b**) *Trichoderma harzianum* (*N*=20 per treatment, Mantel–Cox log rank test, *P*<0.01 compared with untreated control and *P*<0.001 compared with reinfected controls) and (**c**) *Beauveria bassiana* (*N*=20 per treatment, Mantel–Cox log rank test, *P*<0.01 compared with reinfected controls). (**d**) Picture of a representative symbiotic and aposymbiotic egg after 4 days of exposure to *P. lilacinum* spores. Scale bar, 0.5 mm. (**e**) The growth of *P. lilacinum* on the egg has a negative effect on the survival of the larvae during the first days after hatching (*N*=180 per treatment, Cox mixed effects model, *P*<0.001). (**f**) *In vitro* co-cultivation of *B. gladioli* Lv-StA (left) and *P. lilacinum* (right) on potato dextrose agar showing inhibitory activity of *B. gladioli* Lv-StA. Statistically significant differences: **P<0.01 and ***P<0.001. (**a**–**c**,**e**) Estimated survival curves (Kaplan–Meier) and the corresponding standard error are shown.

The specialized localization of the symbionts in the larval and adult stage and the vertical transmission route suggested an important functional role of *Burkholderia* in the insect host. We therefore generated symbiont-free (aposymbiotic) beetles by egg-surface sterilization to evaluate potential differences to their untreated symbiotic counterparts. Notably, aposymbiotic eggs suffered more frequently from fungal infestation, pointing to a protective role of the symbionts. To test this hypothesis, we isolated spores of the most frequently encountered fungal antagonist of *L. villosa* eggs under laboratory conditions, *Purpureocillium lilacinum* (formerly *Paecilomyces lilacinus*), that has been previously reported as an egg entomopathogen^9^ and as a natural enemy of *L. villosa* adults and larvae^10^. Upon exposure to the fungal pathogen, surface-sterilized eggs experienced fungal growth significantly more often and at higher levels than control eggs (Fig. 2a,d and Supplementary Fig. 3). Importantly, reinfection of surface-sterilized eggs with *Burkholderia* symbionts from egg washes or cultured *B. gladioli* Lv-StA significantly reduced fungal infestation, confirming that the absence of the symbionts rather than the surface-sterilization procedure itself was responsible for increased susceptibility to fungal growth (Fig. 2a and Supplementary Fig. 3). The symbionts' protective effect was further corroborated by the significantly higher probability of fungal growth on untreated eggs from aposymbiotic as compared to symbiotic mothers (Supplementary Fig. 4). In addition to *P. lilacinum*, the symbionts also inhibited the growth of the fast-growing soil fungus *Trichoderma harzianum* and the entomopathogen *Beauveria bassiana in vivo*, revealing a generalized antifungal protection by the symbionts (Fig. 2b,c). Although eggs suffering from *P. lilacinum* infection hatched at similar rates as those without fungus (Supplementary Fig. 5a), larvae hatching from infected eggs had significantly lower chances of surviving the first instars, demonstrating that fungal inhibition by the symbionts confers a benefit to the host (Fig. 2e and Supplementary Fig. 5b). Furthermore, the impact of fungal growth on survival varied among the different treatments and was most pronounced for aposymbiotic individuals, suggesting that there may be costs of the symbiosis in the absence of fungal infection (Supplementary Fig. 5b).

### Antibiotic production by *B. gladioli* Lv-StA

**Figure 3:**
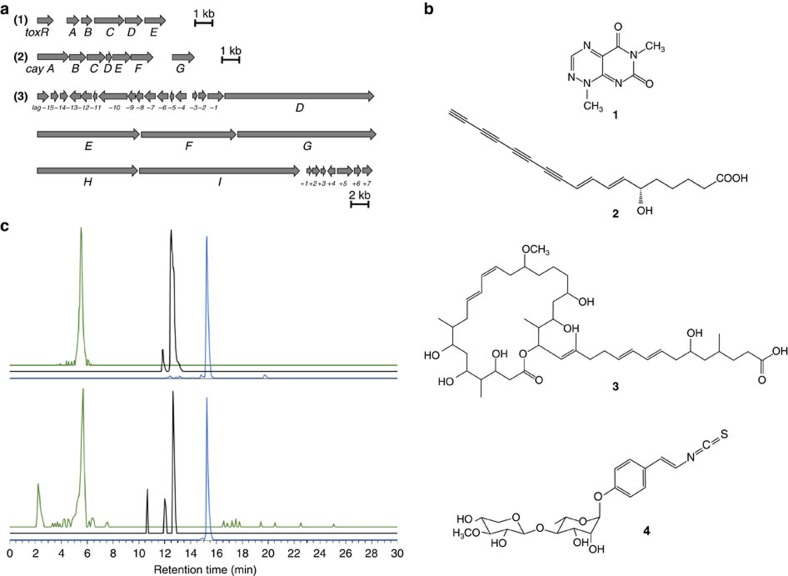
The *Burkholderia* symbionts of *L. villosa* produce several antimicrobial compounds. (**a**) Organization of biosynthetic gene clusters in *B. gladioli* Lv-StA underlying the production of (**1**) toxoflavin (*tox*), (**2**) caryoynencin (*cay*) and (**3**) lagriene (*lag*). (**b**) Chemical structures of toxoflavin (**1**), caryoynencin (**2**), lagriene (**3**) and sinapigladioside (**4**). (**c**) LC-HRESI-MS profiles (extracted mass traces) of toxoflavin (green, *m/z*=194.0669–194.0677), sinapigladioside (black, *m/z*=468.1314–468.1352) and lagriene (blue, *m/z*=779.5265–779.5343) produced by *B. gladioli* Lv-StA *in vitro* (top) and *in vivo*, that is, on *L. villosa* eggs reinfected with *B. gladioli* Lv-StA (bottom). Peak intensity represents relative abundance within each chromatogram, but is not comparable among the green, black and blue profiles. In the egg extracts, toxoflavin was detected in one of the three replicates, whereas lagriene and sinapigladioside were detected in all three replicates. Production of the compounds was independent of the exposure to *P. lilacinum*, and was not detected in the aposymbiotic controls in any of the replicates.

Given that the cultured bacterial symbiont (*B. gladioli* Lv-StA) showed strong antifungal activity *in vitro* (Fig. 2f) and its application *in vivo* fully restored the naturally observed egg protection (Fig. 2a–c), we used this strain to investigate the chemical nature of the symbiont-conferred protection based on whole-genome sequencing, chemical analyses and bioassays. Bioinformatic mining revealed several secondary metabolite biosynthesis gene clusters including those coding for the previously described bioactive compounds toxoflavin^11^ (Supplementary Table 2) and caryoynencin^12^ (Supplementary Table 3), as well as an orphan gene cluster coding for a complex polyketide, homologous to the etnangien biosynthetic assembly line characterized in *Sorangium cellulosum*^13^ (Fig. 3a and Supplementary Tables 4 and 5). High-performance liquid chromatography mass spectrometry (HPLC-MS)-based metabolic profiling of *B. gladioli* Lv-StA culture extracts confirmed the production of the azapteridine toxoflavin (**1**) and the polyyne caryoynencin (**2**), which represent known bioactive metabolites^14^,^15^,^16^,^20^, as well as a polyketide structurally related to etnangien, which we named lagriene (**3**) (Fig. 3b and Supplementary Fig. 6). The structure of lagriene was elucidated using MS and nuclear magnetic resonance (NMR) (Supplementary Figs 7–14). Additionally, an aromatic glycoside was identified in the extracts (Supplementary Fig. 6b), and its structure was fully resolved by MS and NMR (**4**) (Fig. 3b and Supplementary Figs 7,15–20). The latter compound, which we named sinapigladioside, contains an isothiocyanate moiety, a rare structural feature among bacterial metabolites^17^. Similar sulfur-containing structures are typically found in metabolites of plants (mustard oils), mainly of the order Brassicales, where they serve as chemical defence against herbivores and pathogens^18^,^19^. The production of these four compounds by *B. gladioli* Lv-StA suggested them as candidates for the protective effect on the eggs. To evaluate the *in vivo* production of the identified metabolites, we analysed extracts from eggs that were surface sterilized, reinfected with *B. gladioli* Lv-StA and exposed (or not) to *P. lilacinum* spores. Using HPLC-MS, we confirmed the presence of toxoflavin, lagriene and sinapigladioside, implying that the symbionts also produce these antimicrobial agents *in vivo*, without requiring a trigger from the antagonistic fungus (Fig. 3c). Unsurprisingly, caryoynencin was not detected in the extracts, since this highly reactive compound is known to degrade rapidly^12^,^20^. To shed further light on the functional role of the bacterial metabolites, we assessed the activity of the compounds against a number of entomopathogenic microorganisms *in vitro*. Whereas toxoflavin and lagriene possess antibacterial activity (for example, against *Brevibacillus laterosporus* and *Bacillus thuringiensis*, respectively) (Supplementary Note 1), caryoynencin and sinapigladioside displayed antifungal activities against *P. lilacinum* (Supplementary Note 1 and Supplementary Fig. 21). Thus, the beetle symbionts provide a blend of chemical defence compounds against fungal pathogens and potential bacterial antagonists.

### Ancestral plant-associated lifestyle of the symbionts

**Figure 4:**
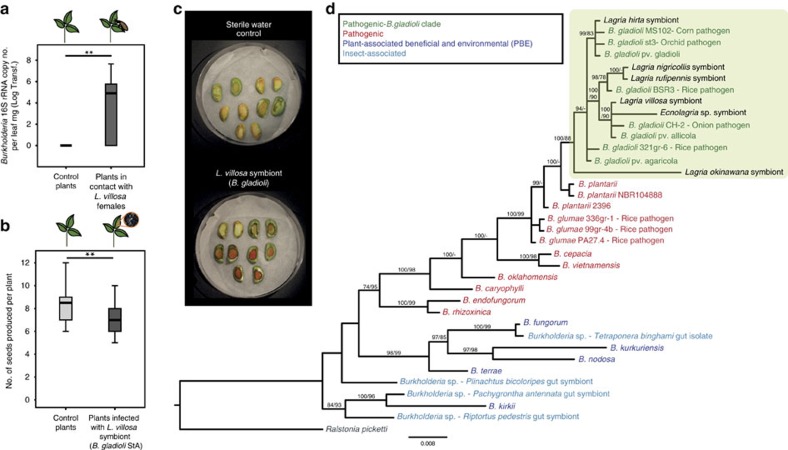
The symbionts of *L. villosa* likely evolved from plant-pathogenic *B. gladioli* and retain their ability to infect a plant host. (**a**) *L. villosa* females transmit *Burkholderia* to soybean plants (control plants *N*=9, *L. villosa*-exposed plants *N*=13; Mann–Whitney *U-*test, *P*<0.01). (**b**) Soybean plants infected with the symbiotic *Burkholderia* from *in vitro* cultures show reduced seed output (*N*=18 for each treatment; Mann–Whitney *U-*test, *P*<0.01). (**c**) Cotyledon assays reveal recognition of microbial elicitors by soybean (red colouration of wounded tissue) upon exposure to symbiotic *B. gladioli*. (**d**) Phylogenetic reconstruction based on Bayesian and approximately maximum likelihood algorithms of selected *Burkholderia* using partial 16S rRNA gene sequences (1,148 bp) showing the placement of Lagriinae-associated *Burkholderia* clustering with plant-pathogenic *B. gladioli*. Posterior probabilities (Bayesian inference) and local support values (FastTree) above 70% are reported at the nodes. References to sequences extracted from public databases and their categorization are listed in Supplementary Table 1. In (**a**, **b**), the centre value of the boxplots represents the median, the boxes denote the interquartile range, and the whiskers represent minimum and maximum values. Statistically significant differences: **P<0.01.

The finding that *B. gladioli* protects an insect was unexpected given that many *B. gladioli* strains are well-known plant pathogens^21^. Hence, we set out to investigate (1) whether the beetle symbionts evolved from plant-pathogenic ancestors, and (2) whether they retained the ability to successfully infect host plants. Characterization of the bacterial symbionts in Lagriinae beetles from Europe (*Lagria hirta*), Brazil (*L. villosa*), Japan (*Lagria nigricollis*, *Lagria rufipennis* and *Lagria okinawana*) and Australia (*Ecnolagria* sp.) revealed the presence of *B. gladioli* in accessory glands of females in all six species (Fig. 4d and Supplementary Table 1). Thus, considering also the morphological description of symbiont-bearing organs in numerous other Lagriinae species^7^, the association with *B. gladioli* is likely ancient and widespread in this beetle subfamily. A 16S rRNA-based phylogeny revealed that the symbionts are interspersed within the monophyletic clade of plant-pathogenic *B. gladioli*, supporting the symbionts' plant pathogenic ancestry (Fig. 4d). The lack of symbiont monophyly indicates that—despite a considerable degree of symbiont specificity and a characterized route for vertical transmission—Lagriinae beetles at least occasionally exchange *B. gladioli* strains with their environment.

### Interaction of the *B. gladioli* symbionts with soybean plants

Experimental exposure of soybean plants to female beetles and subsequent screening for *Burkholderia* indeed revealed the transfer of symbionts to the plant tissue (Fig. 4a). Furthermore, artificial infection of soybean with *B. gladioli* Lv-StA resulted in systemic infection (Supplementary Fig. 22) and reduced seed production compared with water-treated controls (Fig. 4b). Concordantly, cotyledon assays revealed that soybean mounts a defence response against *B. gladioli* Lv-StA (Fig. 4c and Supplementary Fig. 23), showing that the plant recognizes the beetle symbiont as a pathogen. It is conceivable that toxoflavin production by Lv-StA is involved in plant pathogenicity, as has been previously demonstrated for *B. gladioli* and *B. glumae*^22^,^23^. Thus, the beetle is capable of transmitting its symbiotic *B. gladioli* to soybean plants, where the bacteria can reproduce, spread systemically and ultimately impact plant fitness, confirming that the bacteria maintain the potential to interact pathogenically with a plant host, as well as mutualistically with the beetle.

## Discussion

The defensive symbiosis with *Burkholderia* constitutes a potential key innovation in Lagriinae beetles for the protection of the vulnerable and immobile egg stage exposed to the soil environment. Considering that egg-surface contamination is a widespread route for symbiont transmission in insects^24^, this mechanism may have originally evolved for protection or at least may have been reinforced by the additional protective benefit. Symbiont-mediated egg defence is known for some marine crustaceans^25^, and immobile larval stages are symbiotically protected against pathogenic fungi in beewolves^26^ and leaf-rolling weevils^27^, indicating that the protection of immature animals through mutualistic microbes may be a common phenomenon. In this context, the prolific production of a broad spectrum of secondary metabolites^28^ and the ability to engage in pathogenic or mutualistic interactions with a wide range of eukaryotic hosts^29^ may predispose members of the genus *Burkholderia* for defensive symbioses. The *in vitro* and genomics-guided metabolic profiling of the cultured *Lagria* symbiont as well as the chemical analysis of the reinfected beetle eggs revealed that the associated *B. gladioli* can provide a diverse antimicrobial armory. In addition, we provide evidence that secondary metabolite production is important for beetle protection and potentially also for plant pathogenicity. However, the symbiont-mediated production of the four identified compounds and their fitness consequences for the beetle under field conditions remain to be assessed, and at present we cannot completely rule out the possibility that competitive exclusion or additional bioactive metabolites contribute to the symbionts' protective effect on beetle eggs.

Our observation that a protective beetle symbiont evolved from plant pathogenic ancestors and retains the ability to infect the beetle's host plant provides a plausible scenario for the evolutionary switch from pathogenicity to a dual lifestyle. There are many examples of insect-vectored plant pathogens^30^, and some of these indirectly benefit their insect vector by altering plant physiology or suppressing plant defences^30^. However, the Lagriinae symbiosis is exceptional in that the symbionts are not only consistently associated with their insect host, but also provide a direct benefit to the insect that is independent of its plant pathogenic effects. Our findings provide evidence for a transition from a plant pathogenic to an insect mutualistic lifestyle, and describe an ecological setting in which it likely occurred. They also support that such shifts are not necessarily unidirectional, but can be dynamic on both ecological and evolutionary timescales. In addition, our findings highlight arthropod-associated microbes as promising sources for novel bioactive compounds.

## Methods

### Insect collection and rearing

*L. villosa* individuals were collected in soybean and coffee plantations in the localities of Itajú (S21°58′54′′, W48°49′44′′), São Carlos (S21°42′32′′, W47°55′10′′) and Corumbataí (S22°11′02′′,W47°38′53′′) within the state of São Paulo, Brazil, between January and February 2015 (ICMBio authorization no. 45742-1, CNPq process no. 01300.004320/2014-21). Adults were fed with soybean leaves and kept in a greenhouse at 23–26 °C with a natural light regime. Autoclaved water was supplied in centrifuge tubes with cotton, and moist cotton was provided for egg laying. *L. hirta* specimens were collected in Ammerbach-Jena, Germany. *L. rufipennis* specimens collected in Osaka, Japan, were provided by Dr Kimio Masumoto (Sumida-ku Tokyo, Japan). Additional *L. rufipennis* specimens collected in Ibaraki, Japan, were provided by Professor Dr Takema Fukatsu (National Institute of Advanced Industrial Science and Technology, Tsukuba, Japan), who also provided *L. okinawana* specimens collected in Cape Kyan, Okinawa, Japan. *L. nigricollis* specimens collected in Tokushima, Osaka and Kagoshima, Japan, were provided by Dr Kiyoshi Ando (Ehime University, Matsuyama, Japan). A single *Ecnolagria* sp. specimen from NSW Morton National Park, Australia, was provided by Dr Adam Slipinski (Australian National Insect Collection) and Professor Dr Rolf Beutel (Friedrich Schiller University Jena).

### Fluorescence *in situ* hybridization

FISH was carried out on sections of an *L. villosa* larva and female reproductive system, respectively, and on a suspension containing bacteria recovered from the egg surface. The Cy3-labelled *Burkholderia*-specific probe Burk16S (5′-TGCGGTTAGACTAGCCACT-3′) (modified from primer BKH1434Rw)^31^ and the Cy5-labelled general eubacterial probe EUB338 (5′-GCTGCCTCCCGTAGGAGT-3′)^32^ were used for hybridization, and DAPI (4′,6-diamidino-2-phenylindole) for host DNA counterstaining. Embedding, sectioning and FISH were performed as described previously^33^, using a hybridization temperature of 55 °C.

### Three-dimensional reconstruction of a beetle larva

An *L. hirta* larva was fixated in Bouin solution at 4 °C, dehydrated in a graded ethanol series and in isopropanol and embedded using the Epoxy Embedding Medium kit (Sigma-Aldrich, Germany) following the manufacturer's instructions. Hardened epoxy blocks were cut in a rotation microtom (Mikrom HM355S, Thermo-Scientific, Germany) to sections 2 μm thick and stained with Toluidine blue-pyrimidine solution, humidified with xylol and covered with Entellan (Merck, Germany). Section images were acquired in an Axioimager Z1 Microscope (Carl Zeiss, Germany), and the reconstruction was carried out using Amira 5.4.1. software.

### Symbiont cultivation for bioassays and genome sequencing

Live *L. villosa* female adults were placed at –20 °C for 20 min and subsequently surface sterilized by rinsing in 70% ethanol. The paired glandular structures associated to the ovipositor were dissected in sterile phosphate-buffered saline (PBS), and one of these was stored at –80 °C for nucleic acid extraction. The second one was homogenized in 100 μl of sterile PBS and diluted to a factor of 10^−3^, 10^−4^ and 10^−5^. Then, 100 μl of each dilution were plated on Nutrient Agar, R2A Agar (Carl Roth GmbH, Germany) and Actinomycete Isolation Agar (Sigma-Aldrich) and incubated at 30 °C. After 3 days, colonies with distinct morphologies were selected, and part of their biomass was transferred into a lysis solution (67 mM Tris-HCl (pH 8.8), 16.6 mM (NH_4_)_2_SO_4_, 5 mM β-mercaptoethanol, 6.7 mM MgCl_2_, 6.7 μM EDTA (pH 8.0) and 1.7 mM SDS) and kept at 90 °C for 5 min. This suspension containing free DNA was used for a diagnostic PCR with primers specific to the 16S rRNA gene of *Burkholderia*, BKH1434Rw (3′-TGCGGTTAGRCTASCYACT-5′)^31^ and Burk3fwd (3′-CGGCGAAAGCCGGAT-5′; modified from ref. 34). Pure cultures of colonies corresponding to *B. gladioli* Lv-StA were kept as glycerol stocks until further use. Genomic DNA isolation was performed with the QIAGEN Genomic-tip 100/G kit (Qiagen, Hilden, Germany) following the manufacturer's instructions. Genome sequencing was carried out using SMRT (single molecule, real-time) technology provided by Eurofins Genomics, Germany. To identify candidate biosynthesis gene clusters, antiSmash 2.0 (refs 35, 36) and the Artemis genome browser and annotation tools^37^ were used.

### Cultivation and extraction of *B. gladioli* Lv-StA for metabolic profiling

Bacteria were grown in either MGY liquid medium consisting of yeast extract (1.25 g l^−1^) and M9 salts (50 × , part A: 350 g l^−1^ K_2_HPO_4_; 100 g l^−1^ KH_2_PO_4_; part B: 29.4 g l^−1^ tri-Na-citrate-dihydrate; 50 g l^−1^ (NH_4_)_2_SO_4_; 5 g l^−1^ MgSO_4_) and glycerol (10 g l^−1^) (for toxoflavin/lagriene production) or in PDB (Difco) (for caryoynencin/sinapigladioside production) at 30 °C and 110 r.p.m. for 5 or 1 days, respectively. The cultures were extracted with ethyl acetate, dried with sodium sulfate and concentrated under reduced pressure. For LC-MS measurements the extracts were dissolved in 500 μl methanol. For lagriene production, 50 l of MGY medium were inoculated with a 1-day-old bacterial pre-culture (1.5 l in MGY) and incubated at 30 °C for 26 h followed by incubation at 28 °C for 76 h. For sinapigladioside isolation, 3 l of PDB was inoculated and incubated at 30 °C and 110 r.p.m. for 24 h. The extraction was performed as described above.

### Fungal inhibition on eggs and survival assays

A layer of vermiculite substrate was added to 96-well plates and moistened with sterile water. Filter paper discs were then added individually to each well, excluding outermost rows and columns to avoid heterogeneous humidity conditions. A total of 50 fungal (*P. lilacinum*, *T. harzianum* or *B. bassiana*) spores suspended in water were inoculated into each well (Supplementary Table 6).

For the assay with *P. lilacinum*, we initially used 12 different clutches laid by field-collected females. Only the six clutches that hatched were included in the analysis, that is, a total of 720 *L. villosa* eggs (120 eggs per clutch). Eggs from each clutch were divided into four groups of 30 eggs and randomly assigned to four different treatments. For the assays with *T. harzianum* and *B. bassiana*, 80 eggs from the same clutch were used (20 eggs per treatment), respectively. All eggs were placed individually and distributed randomly in relation to treatment in the 96-well plates containing the fungal spores. The first group remained untreated as a control. The three remaining groups were washed in PBS and then surface sterilized by submerging them for 5 min in 90% ethanol, followed by 30 s in 12% NaClO, and a final rinse with sterile water. From the three surface sterilized groups, one (reinfected culture) was reinfected with a PBS suspension (2.5 μl per egg) of symbiotic *B. gladioli* Lv-StA (isolated from *L. villosa*) previously grown in King B medium and adjusted to a concentration of 2 × 10^6^ cells per μl (to achieve a cell number comparable to naturally infected *L. villosa* eggs). The second (reinfected natural) was reinfected with the PBS suspension (2.5 μl per egg) recovered from the egg-washing step previous to sterilization that contained *B. gladioli* and possibly other microbes naturally present on the eggs. Then, 2.5 μl of PBS was added to each egg of the final group (Apo). Plates were stored in closed boxes at 25 °C and monitored daily for visible growth of fungal mycelia on the egg surface. Corresponding treatments were not labelled during monitoring (blind assessment). Hatching rate and survival during the first larval instar and early days of the second instar were also assessed in the assay using *P. lilacinum*.

### Metabolic profiling of *B. gladioli* Lv-StA on *L. villosa* eggs

Half of an *L. villosa* egg clutch was used for each replicate within 36 h after being laid. The eggs were surface sterilized as described in the previous section, and three groups of equal numbers of eggs were assigned each to an individual treatment. The first two groups were reinfected with 2 × 10^6^ *B. gladioli* Lv-StA cells per egg that were previously recovered by centrifuging a pure liquid culture and resuspending in sterile PBS. Eggs from one of these two groups were exposed to *P. lilacinum* spores (250 spores per Petri-dish). The third group remained aposymbiotic as a control. The eggs were kept in a Petri-dish with a layer of moist vermiculite and filter paper at 25 °C. After 3 days, each group was extracted in methanol, and the crude extracts were analysed using liquid chromatography–high-resolution electrospray ionization–mass spectrometry (LC-HRESI-MS) as described below (‘General analytical chemistry procedures' section). The experiment was carried out on three independent *L. villosa* egg clutches.

### Nucleic acid extraction for amplification and sequencing

The accessory glands dissected from adult Lagriinae beetles as described above, whole larvae and eggs (previously subjected to chemical extraction in methanol) were used for nucleic acid isolation. Tissue samples were homogenized in liquid nitrogen and subjected to DNA extraction using the MasterPure complete DNA and RNA isolation Kit (Epicentre). Before protein precipitation, samples were incubated at 37 °C with 4 μl lysozyme (100 mg ml^−1^). The rest of the procedure was carried out following the manufacturer's instructions. Isolated nucleic acids were resuspended in Low TE buffer and stored at –20 °C. The 16S rRNA gene fragment was amplified using general bacterial primers fD1 (5′-AGAGTTTGATCCTGGCTCAG-3′) and rP2 (3′-ACGGCTACCTTGTTACGACTT-5′)^38^ and *Burkholderia*-specific primers BKH1434Rw (3′-TGCGGTTAGRCTASCYACT-5′)^31^ and Burk16S_1F (3′-GTTGGCCGATGGCTGATT-5′). The PCR conditions were 3 min at 94 °C, followed by 32 cycles (bacterial primers) or 42 cycles (*Burkholderia* primers) of 40 s at 94 °C, 60 s at 65 °C (bacterial primers) or 62 °C (*Burkholderia* primers) and 60 s at 72 °C, and a final extension step of 4 min at 72 °C. Purified PCR products were either sequenced directly, or first cloned into *E.coli* StrataClone SoloPack Competent cells (Agilent Technologies, Frankfurt, Germany) using the TOPO TA Cloning Kit (Invitrogen, Darmstadt, Germany) for the cloning reaction. 80μL of each transformation mixture were then plated on LB-ampicillin plates previously spread with 40μL of 2% X-gal (Zymo Research, Freiburg, Germany) and incubated at 37°C overnight. White colonies were used for plasmid insert sequencing using the M13 primer pair from the Strata Clone kit. PCR products were sequenced bidirectionally on an ABI 3730xl capillary DNA sequencer (Applied Biosystems, Foster City, CA, USA). For symbiont quantification in *L. villosa* eggs, larvae and female accessory glands, primers Burk16S_1F (3′-GTTGGCCGATGGCTGATT-5′) and Burk16S_1R (3′-AAGTGCTTTACAACCCGAAGG-5′), which amplify a 172 bp region of *Burkholderia* 16S rRNA, were used for quantitative PCR in a RotorgeneQ cycler (Qiagen) following the protocol described for the Rotor-Gene SYBR Green PCR Kit. PCR conditions were as follows: 95 °C for 10 min, followed by 45 cycles of 95 °C for 10 s and 65 °C for 30 s. A melting curve was subsequently performed with a temperature ramp from 60 to 99 °C within 4.25 min.

### Phylogenetic analyses

*Burkholderia* sequences obtained from accessory glands from females of the six different Lagriinae species were curated manually in Geneious 6.0.5 (ref. 39) and aligned using the SINA alignment software^40^. The phylogenetic reconstruction including a representative or the single available symbiont sequence for each investigated Lagriinae species, and *Burkholderia* references, was based on an approximately maximum likelihood algorithm in FastTree 2.1.8 (ref. 41), using a generalized time reversible model, and on Bayesian inference in MrBayes 3.1.2 (ref. 42) using a HKY substitution model. The Bayesian analysis was run for 100,000 generations, sampling every 100 generations, and a ‘burn-in' of 100 was applied. The phylogenetic reconstruction including the *L. villosa* and *L. hirta* symbiont strains and corresponding references was based on an approximately maximum likelihood algorithm in FastTree 2.1.8 (ref. 41).

### Microbial community analysis

DNA samples from 16 *L. villosa* egg clutches and single accessory glands from 5 adult females were used individually for bacterial community characterization. The 454 pyrosequencing and sequence processing and analyses were carried out as described previously^43^, with minor modifications. Briefly, sequences were obtained from MR DNA (Shallowater, TX, USA) by bacterial tag-encoded FLX amplicon pyroseqencing (bTEFAP) using 16S rRNA primers Gray28F (5′-GAGTTTGATCNTGGCTCA-3′) and Gray519R (5′-GTNTTACNGCGGCKGCTG-3′)^44^ and subsequently analysed in Qiime^45^. After quality filtering, between 10,168 and 26,597 high-quality reads per sample were available for analysis. Sequences were clustered into operational taxonomic units (OTUs) using 97% similarity cutoffs, and one representative sequence per OTU was extracted for taxonomy assignment using the uclust consensus taxonomy assigner. For graphical representation, OTUs corresponding to the same genus were combined, and the percentage of reads in each genus relative to the total reads per sample was plotted.

### Insect-mediated transmission of *Burkholderia* to soybean plants

To test for transmission of *B. gladioli* from the insect host to soybean plants (*Glycine max*, cv. Alligator, N.L. Chrestensen Erfurter Samen- und Pflanzenzucht GmbH, Germany), we confined 18 individual *L. villosa* adults to single leaves and later used quantitative PCR to assess the presence and abundance of live *Burkholderia* in the leaf tissue. In a first group of nine plants, we attached magnetic cages containing a single beetle to two independent leaves per plant. As a control, a second group of nine plants had an empty magnetic cage attached to one leaf. After 3 days, all beetles were removed, and plants were maintained at 21–23 °C with a 16 h light regime for 12 more days until leaves were removed and stored at –80 °C for nucleic acid extraction.

Stored leaves were weighed individually, ground in liquid nitrogen and homogenized. For each sample, a fraction of known weight was separated for RNA extraction and processed using the MasterPure complete DNA and RNA isolation Kit (Epicentre) following the manufacturer's instructions. Reverse transcription was carried out using the Quantitect Reverse Transcription Kit (Qiagen) with *Burkholderia*-specific primers Burk16S_1F (3′-GTTGGCCGATGGCTGATT-5′) and Burk16S_1R (3′-AAGTGCTTTACAACCCGAAGG-5′). The resulting complementary DNA (cDNA) was used for quantitative PCR as described above (‘Nucleic acid extraction, amplification and sequencing' section). Beetles recovered from the experiment were dissected to confirm sex, revealing that 5 out of the original 18 individuals were males. Since adult males lack symbiotic *B. gladioli*, only the 13 replicates involving female beetles were included in the analysis. To test for statistically significant differences in *B. gladioli* titres between *L. villosa*-exposed and control plant leaves, Mann–Whitney *U-*test was carried out in SPSS 17.0.

### Plant fitness effect upon *B. gladioli* infection

A total of 36 soybean plants (*G. max*, cv. 29-I, Semillas Panorama, Colombia) were grown for 28 days before treatment. A single leaflet of the first trifoliate leaf on each plant was wounded in a circular area (0.5 cm diameter) using a robotic device that mimics herbivory damage (MecWorm^46^). Symbiotic *B. gladioli* Lv-StA previously isolated from *L.villosa* as described above were cultured overnight in King B liquid medium at 30 °C and constant shaking (200 r.p.m.) and resuspended in sterile water at a concentration of 10^5^ cells per μl. Half of the plants (*N*=18) were inoculated with 10 μl of the bacterial suspension on the wounded area, and the second half were treated with the same volume of sterile water as a control. Plants were kept at room temperature with a 16 h light regime for 38 days after inoculation. Total seed number was determined for all plants, and tissue samples were recovered from three regions on each plant: (1) the wounded area, (2) a different area on the same leaflet and (3) a leaflet of a younger leaf (not wounded). RNA was extracted from the recovered tissues, and quantitative PCR specific for the 16S rRNA gene of *Burkholderia* was carried out on the corresponding cDNA on a 167 bp fragment using primers Burk16S_StAG_F (5′-CTGAGGGCTAATATCCTTCGGGG-3′) and Burk 3.1_R (5′-TRCCATACTCTAGCTTGC-3′) as described for the horizontal transmission experiment.

### Cotyledon assay

Symbiotic *B. gladioli* Lv-StA from *L. villosa* and *Escherichia coli* K-12 (Agilent Technologies, USA) were cultured overnight in King B liquid medium at 30 °C and continuous shaking (200 r.p.m.). A fraction of the cultured *B. gladioli* cells were killed in 70% ethanol for 5 min. All cultures were centrifuged and resuspended in sterile water. The cotyledon bioassay procedure was based on a previously described protocol^47^ with minor modifications. Briefly, 150 cotyledons from 5-day-old *G. max* seedlings were washed in distilled water, placed in 10% NaClO and submerged in distilled water. Groups of 10 cotyledons, using 3 replicates per treatment, were cut and placed on moist filter paper. Then, 50 μl of bacterial suspension containing 10^6^ cells, sterile water (negative control) or β-glucan (200 μg mg^−1^) (positive control elicitor from the cell wall of the phytopathogen *Phytophtora sojae*) were applied on the wounded area of each cotyledon. After a 24 h incubation period, only cotyledons that retained the liquid (eight cotyledons per treatment) were individually washed in Millipore water, and the content of mixed glyceolin isomers was determined by measuring absorbance at 285 nm.

### General analytical chemistry procedures

Analytical HPLC was performed on a Shimadzu LC-10Avp series HPLC system consisting of an autosampler, high-pressure pumps, column oven and photodiode array detector. HPLC conditions were as follows: C18 column (Eurospher 100-5, 250 × 4.6 mm) and gradient elution (MeCN/0.1% (*v/v*) trifluoroacetic acid (TFA) 0.5/99.5 in 30 min to MeCN/0.1% (*v/v*) TFA 100/0, MeCN 100% for 10 min), flow rate 1 ml min^−1^. Preparative HPLC was performed on a Shimadzu LC-8a series HPLC system with photodiode array detector. LC-MS measurements were performed using an Exactive Orbitrap High Performance Benchtop LC-MS with an electrospray ion source and an Accela HPLC system (Thermo Fisher Scientific, Bremen). HPLC conditions were as follows: C18 column (Betasil C18 3 μm 150 × 2.1 mm) and gradient elution (MeCN/0.1% (*v/v*) HCOOH (H_2_O) 5/95 for 1 min, going up to 98/2 in 15 min, then 98/2 for another 3 min; flow rate 0.2 ml min^−1^). For tandem mass spectrometry measurements, a Q Exactive Orbitrap mass spectrometer with an electrospray ion source (Thermo Fisher Scientific) was used. NMR spectra were recorded on a Bruker AVANCE III 600 MHz instrument equipped with a Bruker cryo platform. Spectra were normalized to the residual solvent signals. The infrared spectra were recorded on a JASCO FT/IR-4100 type A.

### Isolation of bioactive compounds

The crude extract was defatted with hexane and fractionated by size exclusion chromatography with Sephadex LH20 using MeOH as eluent. Final purification of compound **3** (lagriene) was achieved by preparative HPLC using a Phenomenex Synergi 4 μm Fusion-RP80A column (250 × 21.2 mm) with a flow rate of 10 ml min^−1^ and a gradient method (MeCN/0.01 TFA (H_2_O, *v/v*) 40/60 for 5 min, going up to 75/25 in 25 min and then increasing to 100% MeCN in 5 min). For compound **1** (toxoflavin), the following HPLC gradient was applied: MeCN/0.01 TFA (H_2_O, v/v) 1/99 for 5 min and going up to 40/60 in 25 min. Compound **4** (sinapigladioside) was isolated from the crude extract by size exclusion chromatography with Sephadex LH20 using 83% MeCN as an eluent followed by preparative HPLC using a Nucleodur VP 250 × 21, C18 HTec, 5 μm with a flow rate of 10 ml min^−1^ and a gradient method (MeCN/0.01 TFA (H_2_O, *v/v*) 30/70 for 5 min and going up to 100/0 in 20 min). Caryoynencin (**2**) was identified by LC-HRESI-MS and comparison with an authentic reference.

### Structure elucidation of lagriene and sinapigladioside

For compound **3** (lagriene), a molecular formula of C_44_H_74_O_11_ was deduced from HRESI-MS measurements. ^13^C and DEPT135 spectra revealed the presence of three quaternary, 22 methine and 13 methylene and 6 methyl carbon atoms. The proton and carbon NMR data indicated the structural relatedness to etnangien^48^. Analysis of the H,H-correlation spectroscopy (H,H-COSY) and the heteronuclear multiple bond correlation (HMBC) couplings identified the backbone of **3** (Supplementary Fig. 7). A coupling constant of *J*_*H,H*_=11 Hz for the protons H-26/H-27 disclosed the *Z* configuration of the respective double bond, whereas all other double bonds were found to be in *E* configuration (*J*_*H,H*_=15 Hz). HMBC coupling of H-16 and C-38 indicated the position of cyclization. For compound **4** (sinapigladioside), a molecular mass of *m/z* 468.1334, amu (M–H)^−^ and a molecular formula of C_21_H_26_NO_9_S (calcd. 468.1334) was determined by HRESI-MS. The number of carbon atoms was corroborated by ^13^C NMR analysis and the multiplicity was assigned by DEPT135 measurements. Proton and carbon NMR data revealed the presence of a *para*-substituted aromatic compound. HMBC couplings of H-8 and C-4 and H-7 and C-3, C-4 and C-6 disclosed the connection of the vinyl moiety. A coupling constant of *J=*13.9 Hz for H-7/H-8 pointed to the *E* configuration of the double bond. HRESI-MS data and a characteristic band in the infrared spectrum (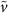 2104, cm^−1^) suggested the presence of an isothiocyanate residue. This assumption was corroborated by an HMBC coupling of H-8 with the quaternary carbon C-9. Analysis of the H,H-COSY spectra revealed the spin systems of the sugar moieties and HMBC couplings of the anomeric protons H-1′ to C-1 and H-1′′ to C-4′, respectively, established their connectivity. An HMBC coupling of the *O*-methyl proton H-3′′-OMe with C-3′′ revealed the position of the methyl group. The nuclear Overhauser effect measurements finally elucidated the sugar moieties as rhamnose and *O*-methyl xylose, respectively. The NMR data are also in good agreement with literature data^49^.

### Antimicrobial bioassays

The antifungal activity of the metabolites was studied by agar diffusion tests. A total of 50 μl of a solution of the respective compound (1 mg ml^−1^ in methanol as a stock solution and respective dilutions) were filled in agar holes of 9 mm diameter (potato dextrose agar, seeded with a spore suspension). After incubation at 30 °C for 24 h, the inhibition zone was measured. Antibacterial activity was tested as described before^50^. Compounds were tested against *Bacillus thuringiensis*, *Brevibacillus laterosporus*, *P. lilacinum* and (if enough material was available) *Bacillus subtilis*, *Staphylococcus aureus*, *Escherichia coli*, *Pseudomonas aeruginosa*, *Staphylococcus aureus*, *Enterococcus faecalis*, *Mycobacterium vaccae*, *Sporobolomyces salmonicolor*, *Candida albicans*, *Penicillium notatum* and *Aspergillus fumigatus* (see Supplementary Table 6 for information on the strains used). Given the high instability of caryoynencin^12^,^20^, antimicrobial assays for this compound were carried out with an authentic caryoynencin fraction derived from a previous study^12^.

### Statistical analyses

Cox mixed effects models with a random intercept per clutch were used to analyse the effect of treatment on *P. lilacinum* growth on eggs, as well as the effect of treatment and fungal growth at the egg stage on the survival of early instar larvae, assuming a Gaussian distribution for the random effects. These statistical analyses were carried out in R 2.14.1. using the coxme package^51^. For *T. harzianum* and *B. bassiana*, the effect of treatment on fungal growth on the eggs was assessed using Mantel-Cox log rank tests in SPSS 17.0. Growth probability of all fungi and larval survival probability (*P. lilacinum* assay) were plotted based on Kaplan–Meier models using the rms package in R 2.14.1 (ref. 52). Generalized linear mixed models with a Poisson distribution were used to analyse fungal growth level and hatching rate using the lme4 package in R 2.14.1 (ref. 53), and overdispersion was calculated manually to test for the validity of the distribution family.

*Burkholderia* abundance in plants upon exposure to the insects, and seed output following *Burkholderia* infection, were analysed using the Mann–Whitney *U*-test in SPSS 17.0. Similar distribution for seed output data was confirmed using a two-sample Kolmogorov–Smirnov test. The quantitative PCR data on *Burkholderia* abundance in different plant locations upon infection were analysed in an analysis of variance and Tukey's *post hoc* tests after confirming the normal distribution of the data.

For the cotyledon assay, the absorbance data were not normally distributed, and thus a Kruskall–Wallis test with Dunn's *post hoc* test was carried out on the in SPSS 17.0. Two-sided tests were used in all cases.

### Data availability

The 16S rDNA nucleotide sequences of *Burkholderia* symbionts have been deposited in the GenBank nucleotide database with accession numbers KT888026 to KT888030, and KU358660 to KU358661. Nucleotide sequences for the toxoflavin, caryoynencin and lagriene gene clusters have been deposited in the European nucleotide archive with accession numbers LT797825 to LT797838. Raw 454 sequencing data corresponding to bacterial 16S rDNA amplicons from *L. villosa* female accessory glands and egg clutches have been deposited in the NCBI Sequence Read Archive with accession codes SAMN04364624 to SAMN04364628, and SAMN04510296 to SAMN04510311, respectively (within BioProject PRJNA306502). The authors declare that all other data supporting the findings of this study are available within the paper and its Supplementary Information files, or from the corresponding authors on request.

## Additional information

**How to cite this article:** Flórez, L. V *et al*. Antibiotic-producing symbionts dynamically transition between plant pathogenicity and insect-defensive mutualism. *Nat. Commun.* **8**, 15172 doi: 10.1038/ncomms15172 (2017).

**Publisher's note:** Springer Nature remains neutral with regard to jurisdictional claims in published maps and institutional affiliations.

## Supplementary Material

Supplementary Information Supplementary Note, Supplementary Figures, Supplementary Tables and Supplementary References
