# Supplementary material for: Antibiotic-producing symbionts dynamically transition between plant pathogenicity and insect-defensive mutualism
Source: Nat Commun. 2017 Apr 28;8:15172. doi: 10.1038/ncomms15172 (PMC5414355; doi:10.1038/ncomms15172)
Supplement: Supplementary Information — Supplementary Note, Supplementary Figures, Supplementary Tables and Supplementary References [file ncomms15172-s1.pdf]

Supplementary Note 1. Physicochemical data and antimicrobial activity of the symbiont-produced compounds

Toxoflavin (1)

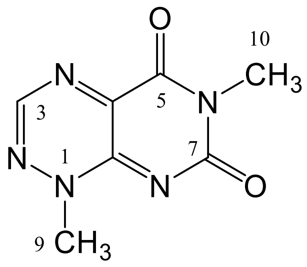

ESI(+) *m/z* 194 $(M+H)^+$, HRESI(+)-MS *m/z* 194.0674 (calcd. for $C_7H_8N_5O_2$ 194.0673)

UV (PDA): λ_{max}= 258, 397 nm

NMR: d_6-DMSO, ^1H NMR 600 MHz, ^{13}C NMR 150 MHz

Carbon	^{13}C	^1H (mult., *J* in Hz)
**3**	144.7	8.96 (s)
**4a**	146.5	-
**5**	154.1	-
**7**	159.0	-
**8a**	150.9	-
**9**	42.4	3.94 (s)
**10**	28.2	3.24 (s)

Antimicrobial activity (1 mg mL^{-1}): An inhibition zone of 22 mm against *Brevibacillus laterosporus* was measured (procedure described in Methods section, *Antimicrobial bioassays*).

Caryoynencin (2)

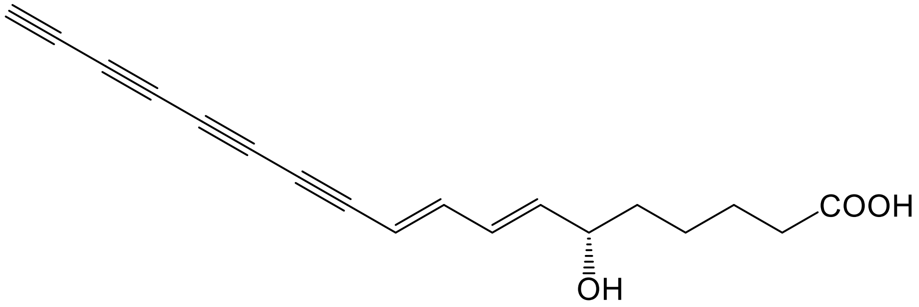

ESI(-) *m/z* 279 $(M-H)^-$, HRESI(-)-MS *m/z* 279.1031 (calcd. for $C_{18}H_{17}O_3$ 279.1027)

UV (PDA): λ_{max}= 294, 239, 280, 268, 358 nm.

Antimicrobial activity: active against *P. lilacinum* (see Methods,*Antimicrobial bioassays*) (Supplementary Fig. 21b).

Lagriene (3)

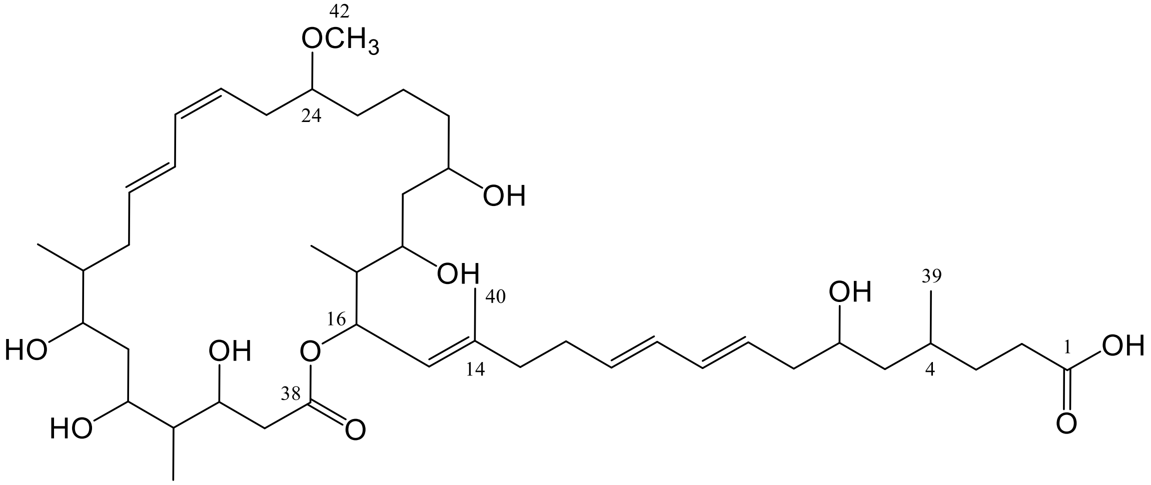

ESI(-) *m/z* 777 $(M-H)^-$, HRESI(-)-MS *m/z* 777.5172 (calcd. for $C_{44}H_{73}O_{11}$ 777.5158)

UV (PDA): λ_{max}= 231 nm

IR-spectrum:

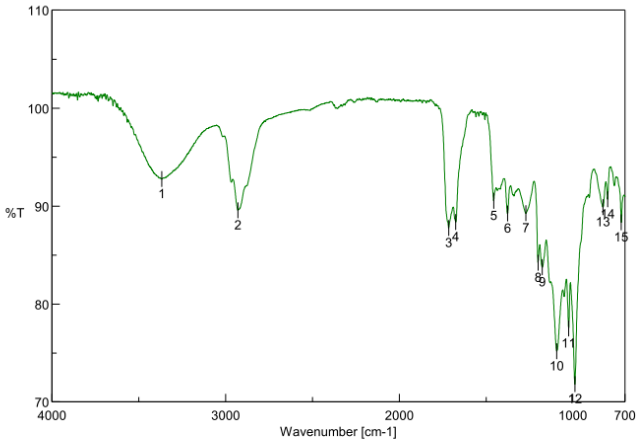

NMR: d_6-DMSO, ^{1}H NMR 600 MHz, ^{13}C NMR 150 MHz

**Carbon**	**^{13}C**	**^{1}H (mult., *J* in Hz)**
**1**	174.1	-
**2**	31.4	2.16 (m)
**3**	32.4	1.47 (m)<br>1.32 (m)
**4**	28.2	1.60 (m)
**5**	43.8	1.26 (m)<br>1.03 (m)
**6**	67.5	3.49 (m)
**7**	41.5	2.08 (m)
**8**	129.3	5.55 (m)
**9**	131.6	5.95 (m)
**10**	130.7	5.95 (m)
**11**	131.2	5.48 (m)
**12**	30.2	2.27 (m)<br>2.11 (m)
**13**	38.9	2.06 (m)<br>2.02 (t, *7.2*)
**14**	139.1	-
**15**	122.9	5.00 (d, *9.5*)
**16**	72.1	5.26 (m)
**17**	41.8	1.78 (m)
**18**	66.5	3.77 (s)
**19**	39.3	1.45 (m)<br>1.32 (m)
**20**	66.8	3.62 (m)
**21**	38.3	1,48 (m)
**22**	20.6	1.35 (m)<br>1.21 (m)
**23**	31.9	1.36 (m)
**24**	79.6	3.21 (m)
**25**	30.1	2.38 (m)<br>2.27 (m)
**26**	125.1	5.26 (m)
**27**	130.1	6.00 (m)
**28**	126.6	6.29 (m)

**29**	133.6	5.72 (m)
**30**	36.5	2.18 (m)
**31**	37.7	1.48 (m)
**32**	71.7	3.53 (m)
**33**	39.3	1.45 (m)<br>1.32 (m)
**34**	69.2	3.84 (m)
**35**	43.6	1.33 (m)
**36**	68.6	3.81 (m)
**37**	39.6*	2.45 (m)<br>2.15 (m)
**38**	170.4	-
**39**	18.8	0.80 (m)
**40**	16.6	1.68 (s)
**41**	10.5	0.71 (d, *7.0*)
**42**	55.6	3.22 (s)
**43**	13.1	0.79 (m)
**44**	9.7	0.76 (d, *7.0*)

* signal overlapping with solvent signal

Antimicrobial activity (1 mg mL^{-1}): Inhibition zones of 23 mm against *Bacillus thuringiensis*, 30 mm against *Mycobacterium vaccae*, 19 mm against vancomycin-resistant *Enterococcus faecalis,* and 18 mm against methicillin-resistant *Staphylococcus aureus* were measured (procedure described in Methods section, *Antimicrobial bioassays*).

Sinapigladioside (4)

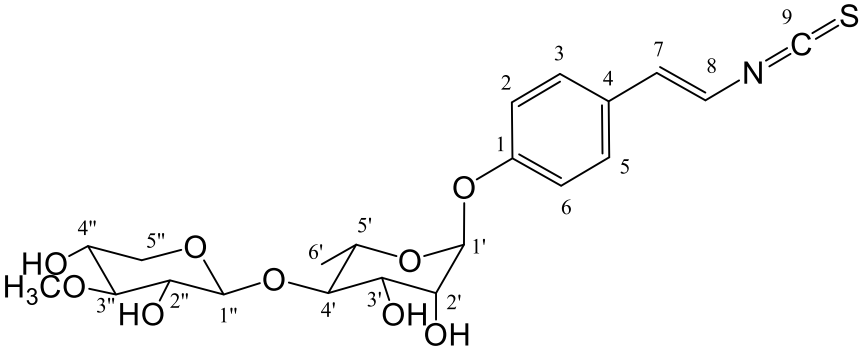

ESI(-) *m/z* 468 $(M-H)^-$, HRESI(-)-MS *m/z* 468.1334 (calcd. for $C_{21}H_{26}NO_9S$ 468.1334)

UV (PDA): λ_{max}= 312 nm.

IR-spectrum:

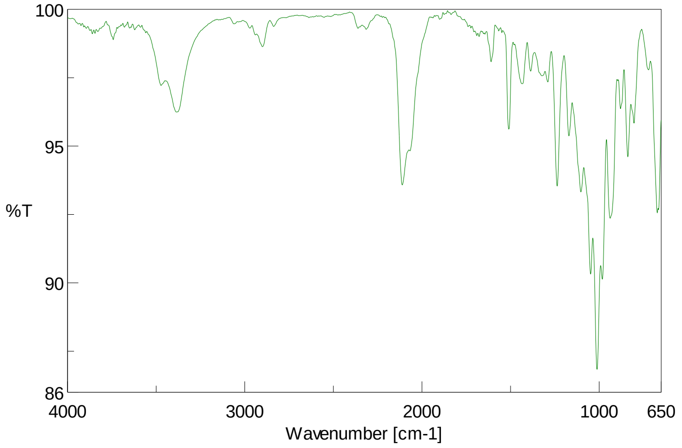

NMR: d_6-DMSO, ^{1}H NMR 600 MHz, ^{13}C NMR 150 MHz

**Carbon**	**^{13}C**	**^{1}H (mult., *J* in Hz)**
**1**	156.4	-
**2**	116.7	7.04 (d, *8.8*)
**3**	128.0	7.43 (d, *8.8*)
**4**	127.3	-
**5**	128.0	7.43 (d, *8.8*)
**6**	116.7	7.04 (d, *8.8*)
**7**	131.7	6.86 (d, *13.9*)
**8**	114.0	7.00 (d, *13.9*)
**9**	131.5	-
**1’**	97.5	5.46 (d, *1.6*)
**2’**	69.3	3.92 (m)
**3’**	68.9	3.75 (m)
**4’**	81.8	3.48 (m)
**5’**	67.9	3.59 (m)
**6’**	17.3	1.20 (d, *6.2*)
**1’’**	104.0	4.25 (d, *7.8*)
**2’’**	72.7	3.10 (m)
**3’’**	86.2	2.93 (t, *9.0*)
**4’’**	68.8	3.42 (m)
**5’’**	65.7	3.75 (m)<br>3.17 (t, *10.9*)
**3’’-OCH_3**	60.0	3.50 (s)
**2’-OH**	-	5.25 (d, *4.3*)
**3’-OH**	-	4.32 (d, *2.5*)
**2’’-OH**	-	5.35 (d, *5.4*)
**4’’-OH**	-	5.15 (d, *5.5*)

Antimicrobial activity (1 mg mL^{-1}): Inhibition zones of 30 mm against *P. lilacinum*, 32 mm against *Aspergillus fumigatus* and 25 mm against *Penicillium notatum* were measured (procedure described in Methods section, *Antimicrobial bioassays*) (Supplementary Fig. 21a).

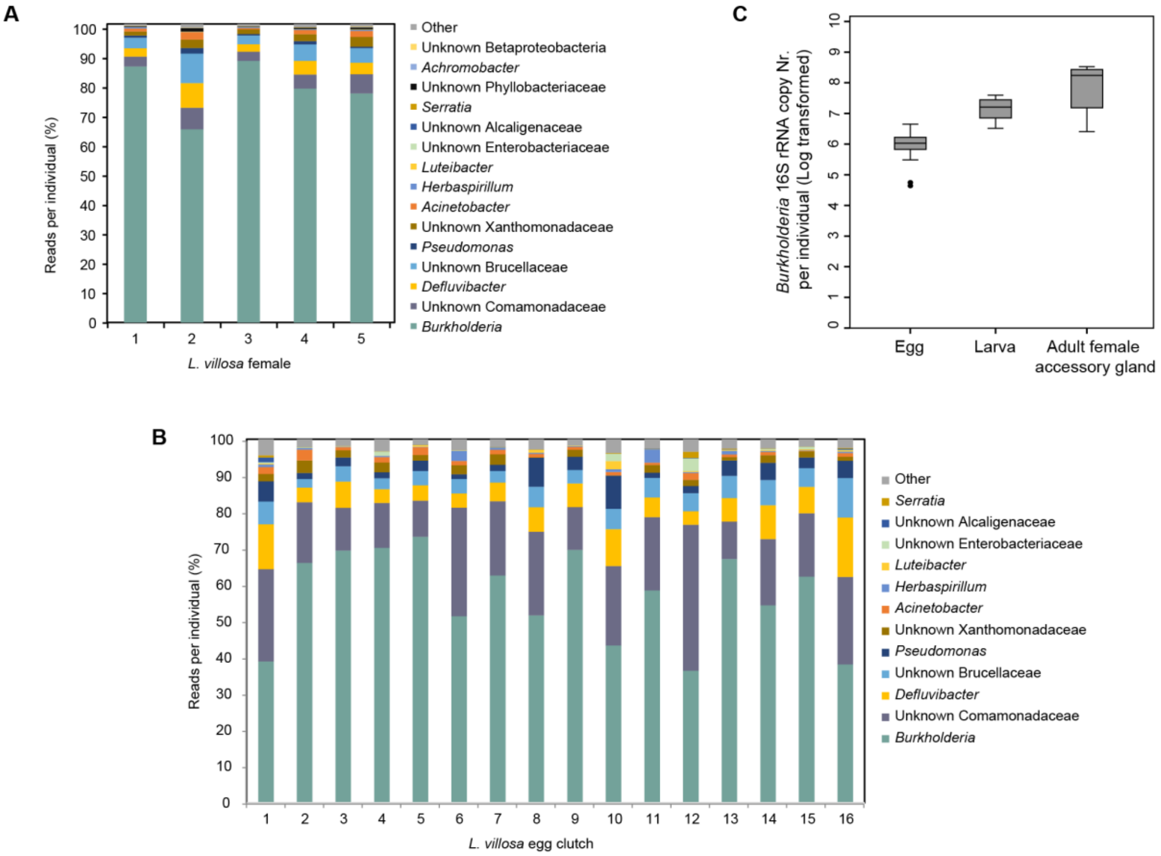

Supplementary Fig. 1. *Burkholderia* is consistently the most abundant taxon in *Lagria villosa* eggs, larvae and the accessory glands associated with the reproductive tract of adult females. Microbial composition as revealed by 454 pyrosequencing of partial 16S rRNA gene sequences carried out on (**A**) the accessory glands of six field-collected *L. villosa* females and (**B**) 16 egg clutches laid by field-collected *L.villosa* females, based on 97% similarity OTU clustering as described in the methods section (Microbial community analysis); (**C**) Quantification of *Burkholderia* symbionts by qPCR using a 172 bp region of the 16S rRNA gene as described in the methods section (Horizontal Transmission experiment) in 15 egg clutches (abundance per individual egg is represented), six larvae between 32 and 43 days old, and eight accessory glands from adult females (abundance for a single gland per individual is represented). The center value of the boxplots represents the median, and the whiskers denote minimum and maximum values.

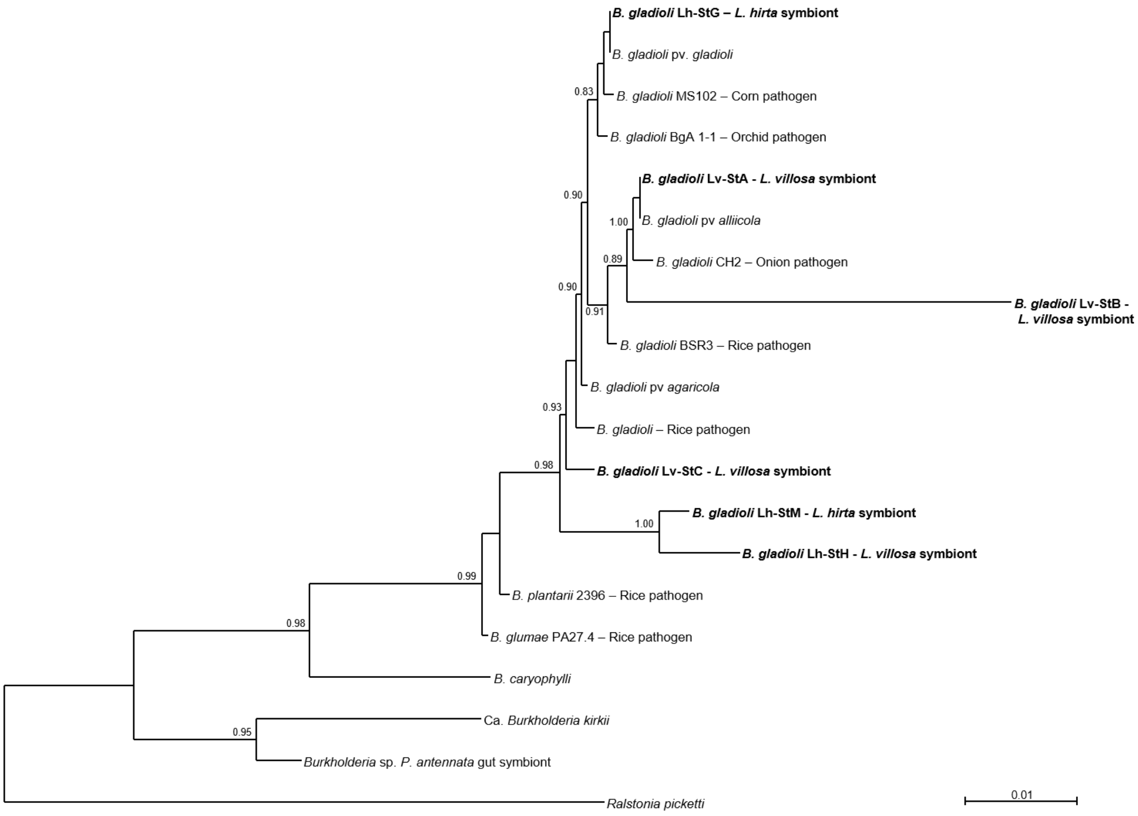

Supplementary Fig. 2. *L. villosa* beetles, as well as the related species *L. hirta*, carry at least three symbiotic *B. gladioli* strains. Phylogenetic reconstruction based on an approximately-maximum-likelihood algorithm of selected *Burkholderia* using partial 16S rRNA gene sequences (1,169 bp), showing the placement of the *L. villosa* - associated strains relative to other *Burkholderia*. Local support values above 0.7 are reported at the nodes. References to sequences extracted from public databases are listed in Supplementary Table 1.

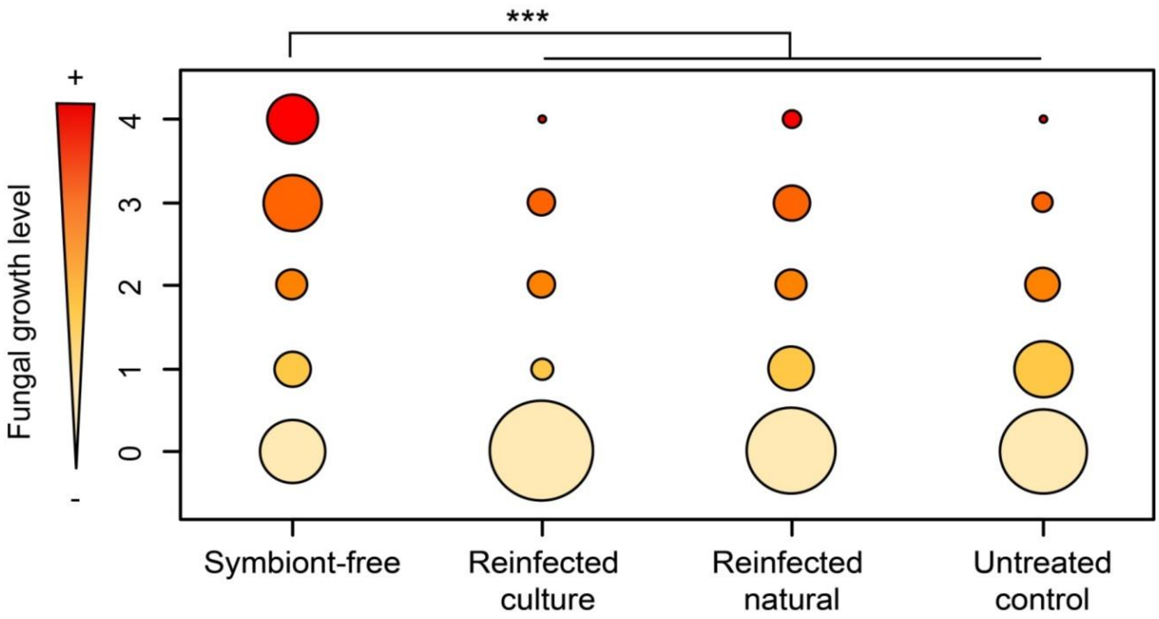

Supplementary Fig. 3. When present, *P. lilacinum* reaches higher biomass on *L. villosa* eggs in the absence of the *Burkholderia* symbionts. Fungal growth was estimated qualitatively during blind monitoring of 720 eggs (180 eggs per treatment from 6 independent clutches) as described in the methods section (*Fungal inhibition on eggs and survival assays*), assigning the level of growth to one of the following categories (0 = no visible growth, 1 = minor growth directly on surface and barely noticeable, 2 = multiple mycelia in contact with surface, 3 = considerable growth on surface, 4 = surface completely covered by mycelia). For statistical analysis, a generalized linear mixed model with a Poisson distribution and clutch as random factor was used (***$p < 0.001$). The radius of each circle corresponds to the number of eggs in the respective category.

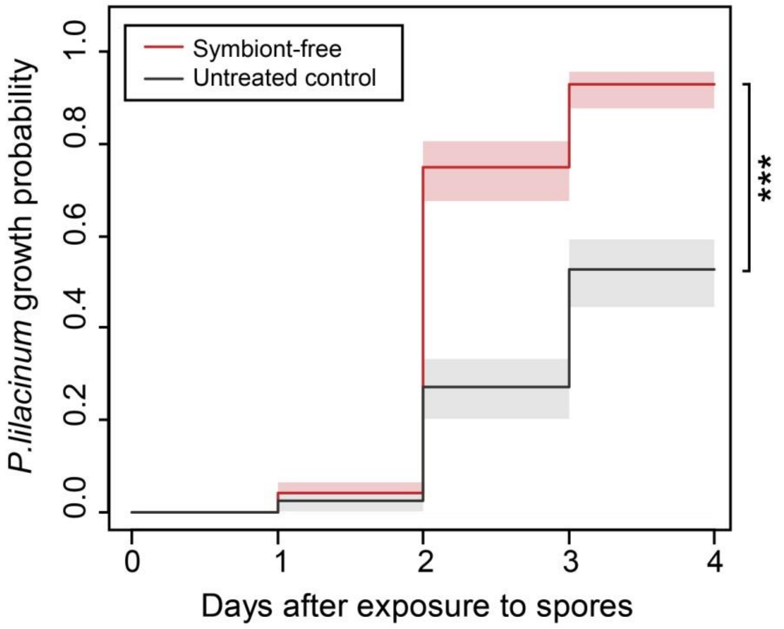

Supplementary Fig. 4. Eggs laid by symbiont-free *L. villosa* females are less protected against *P. lilacinum* fungal growth in comparison to their symbiotic counterparts. Symbiont-free females were obtained by rearing from surface-sterilized eggs, while symbiotic females were taken from the normal beetle culture. For each treatment, six clutches and 30 eggs per clutch were tested as described in the methods section excluding reinfection procedures (*Fungal inhibition on eggs and survival assays*). For statistical analysis, a Cox mixed effects model including clutch as a random factor was used (***$p < 0.001$). Estimated survival curves (Kaplan-Meier) and the corresponding standard error are plotted.

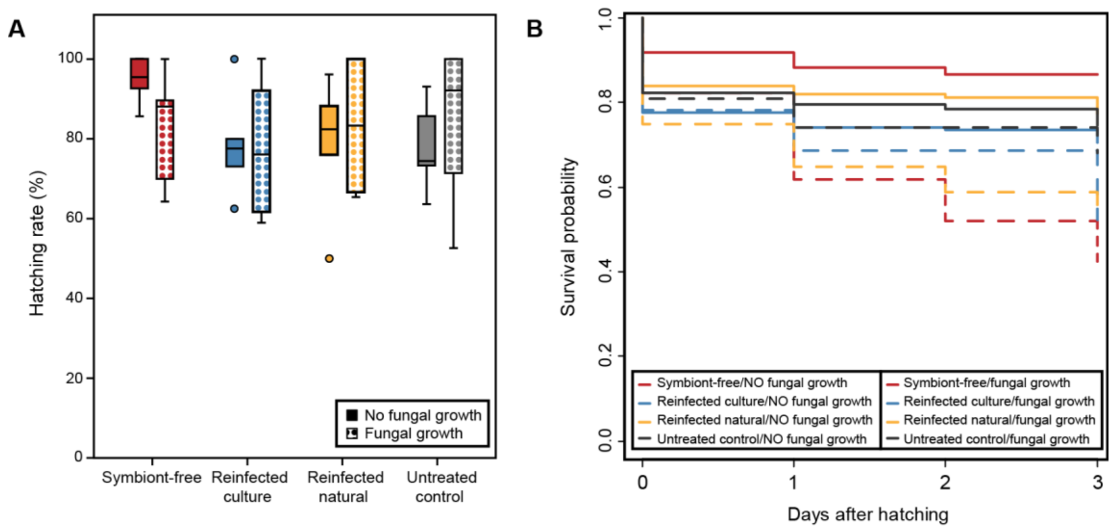

Supplementary Fig. 5. The detrimental effect of *P. lilacinum* growth on *L. villosa* eggs does not significantly reflect on hatching rate, but it causes increased larval mortality in the first days after hatching. (**A**) There was no statistically significant effect of either treatment or fungal growth level on hatching rate of the six egg clutches (Generalized linear mixed model with a Poisson distribution, $p > 0.05$). The center value of the boxplots represents the median, the whiskers denote minimum and maximum values, and circles represent outliers (more than 1.5 interquartile ranges from nearest box edge). (**B**) Fungal growth on the eggs has a negative effect on the survival of the larvae during the first days after hatching and affects individuals from the treatments differently, with aposymbionts showing the most pronounced effect (Cox Mixed-Effects Model; Fungus, $p < 0.001$; Treatment:Fungus, $p < 0.05$). Survival curves based on Kaplan-Meier estimates are plotted. For both (**A**) and (**B**), sample sizes were as follows: Symbiont-free: no fungal growth 60, fungal growth 118; Reinfected culture: no fungal growth 147, fungal growth 32; Reinfected natural: no fungal growth 111, fungal growth 68; Untreated control: no fungal growth 107, fungal growth 73.

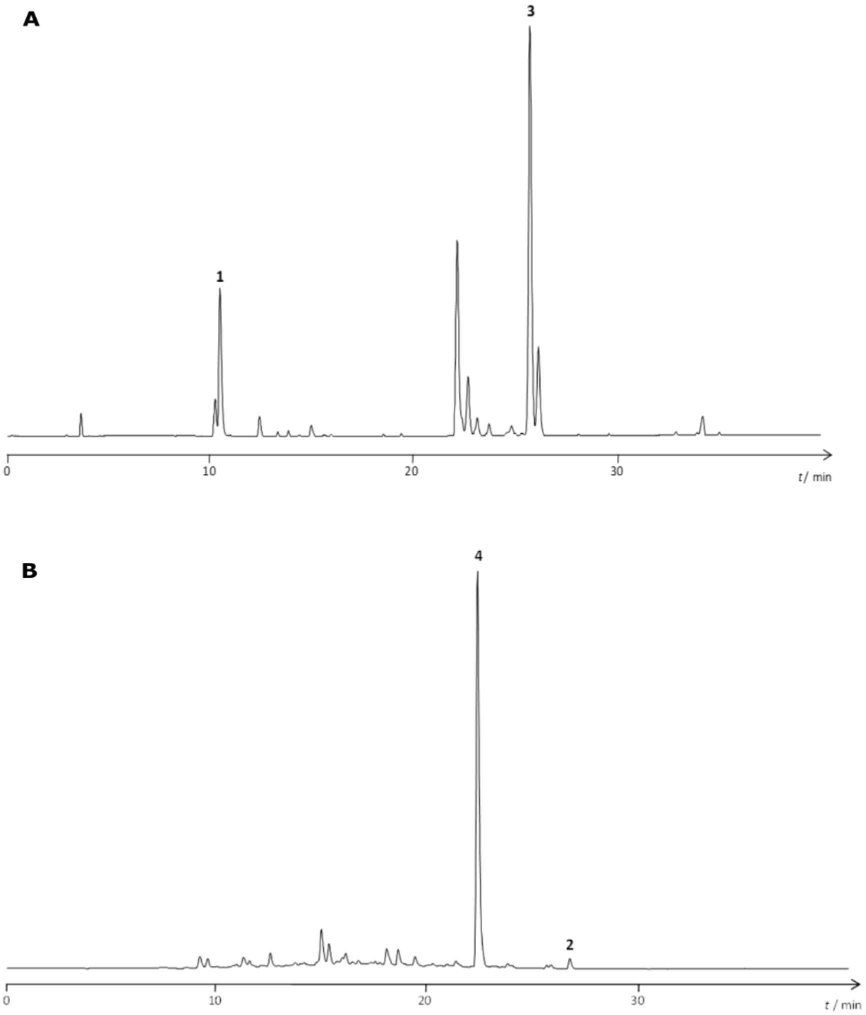

Supplementary Fig. 6. HPLC profiles of crude extracts of *B. gladioli* Lv-StA liquid cultures indicating the production of compounds (1-4) *in vitro*. (**A**). Chromatogram (PDA total scan) of the crude extract of *B. gladioli* Lv-StA cultured on MGY medium. Numbers indicate production of toxoflavin (**1**) and lagriene (**3**). (**B**) HPLC profile (294 nm) of the crude extract of *B. gladioli* Lv-StA cultured on PDB medium. Numbers indicate production of caryoynencin (**2**) and sinapigladioside (**4**).

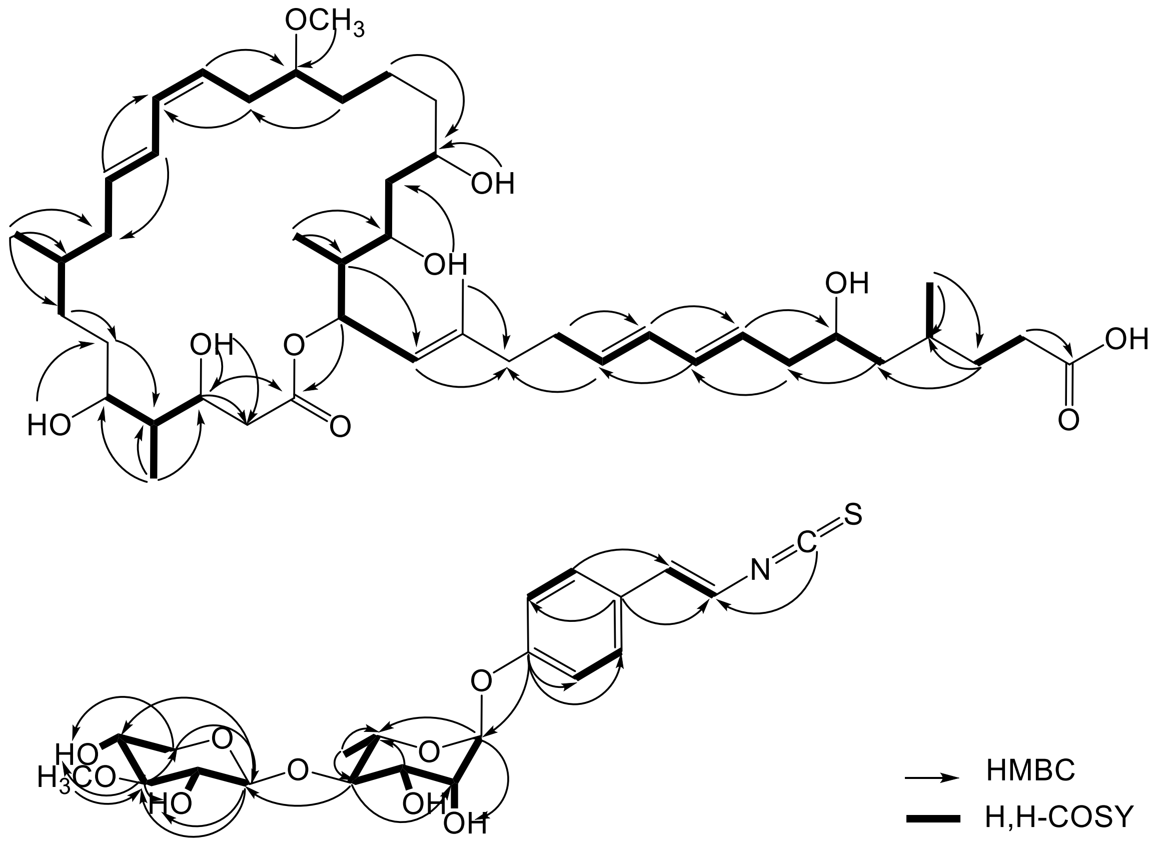

Supplementary Fig. 7. Key 2D NMR couplings of lagriene (3) and sinapigladioside (4).

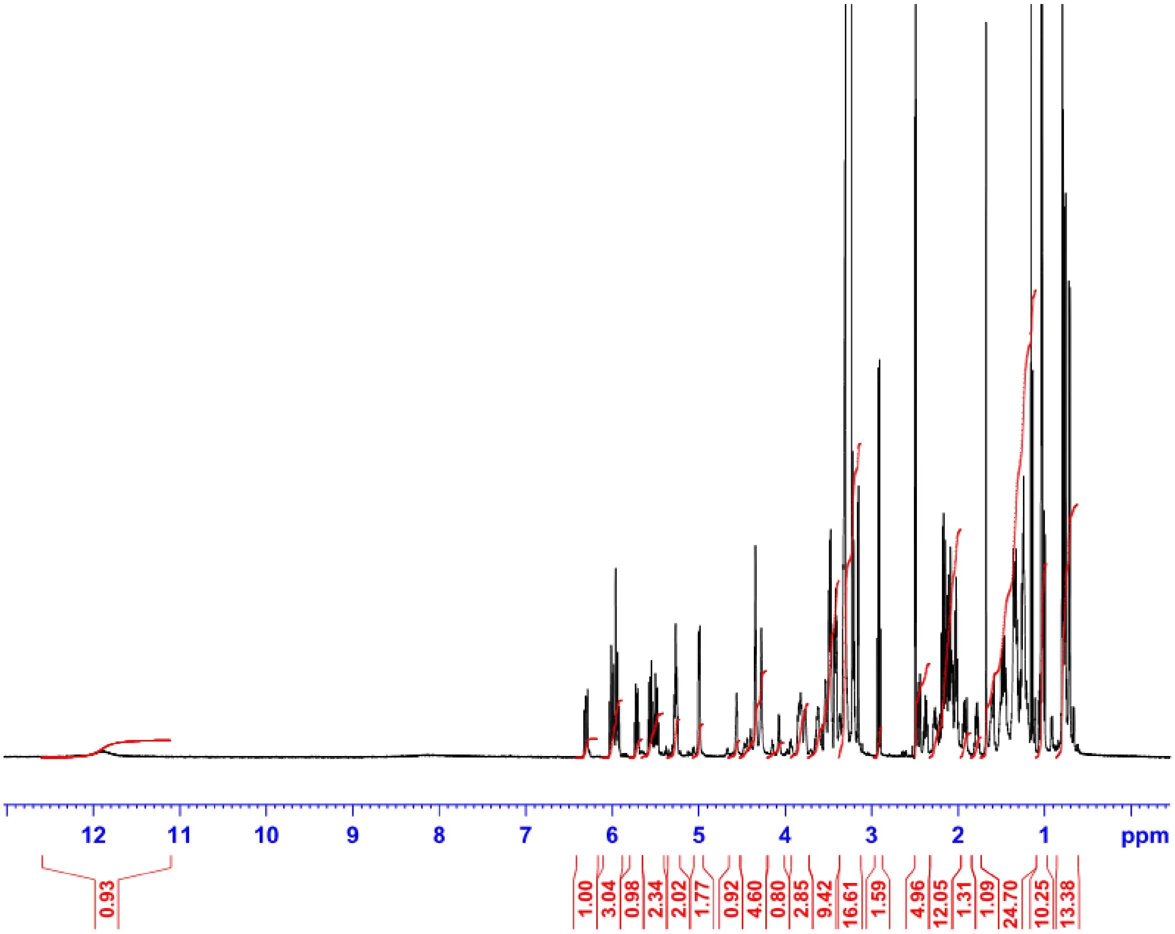

Supplementary Fig. 8. ^{1}H NMR spectrum of lagriene (3).

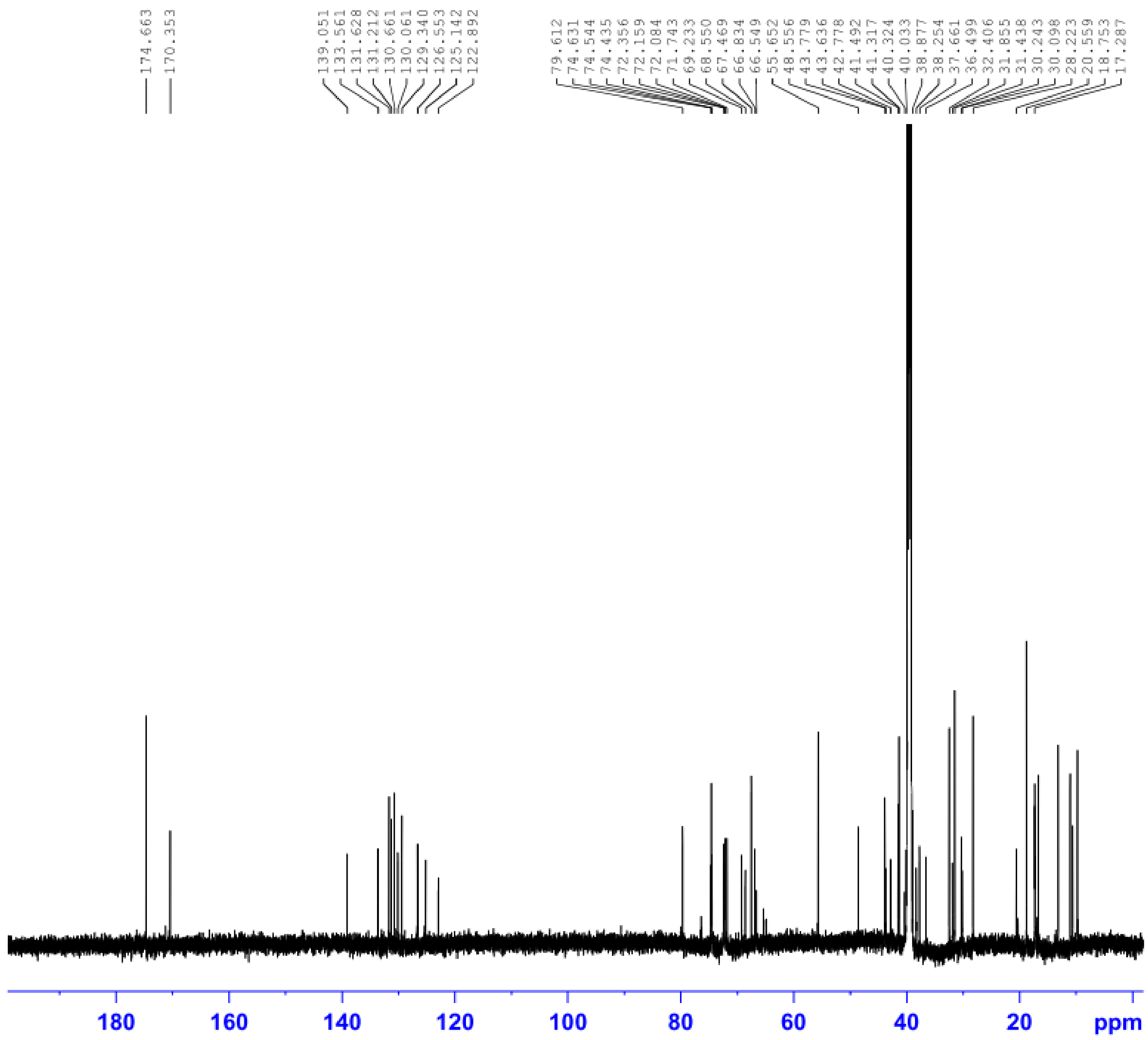

Supplementary Fig. 9. ^{13}**C NMR spectrum of lagriene (3).**

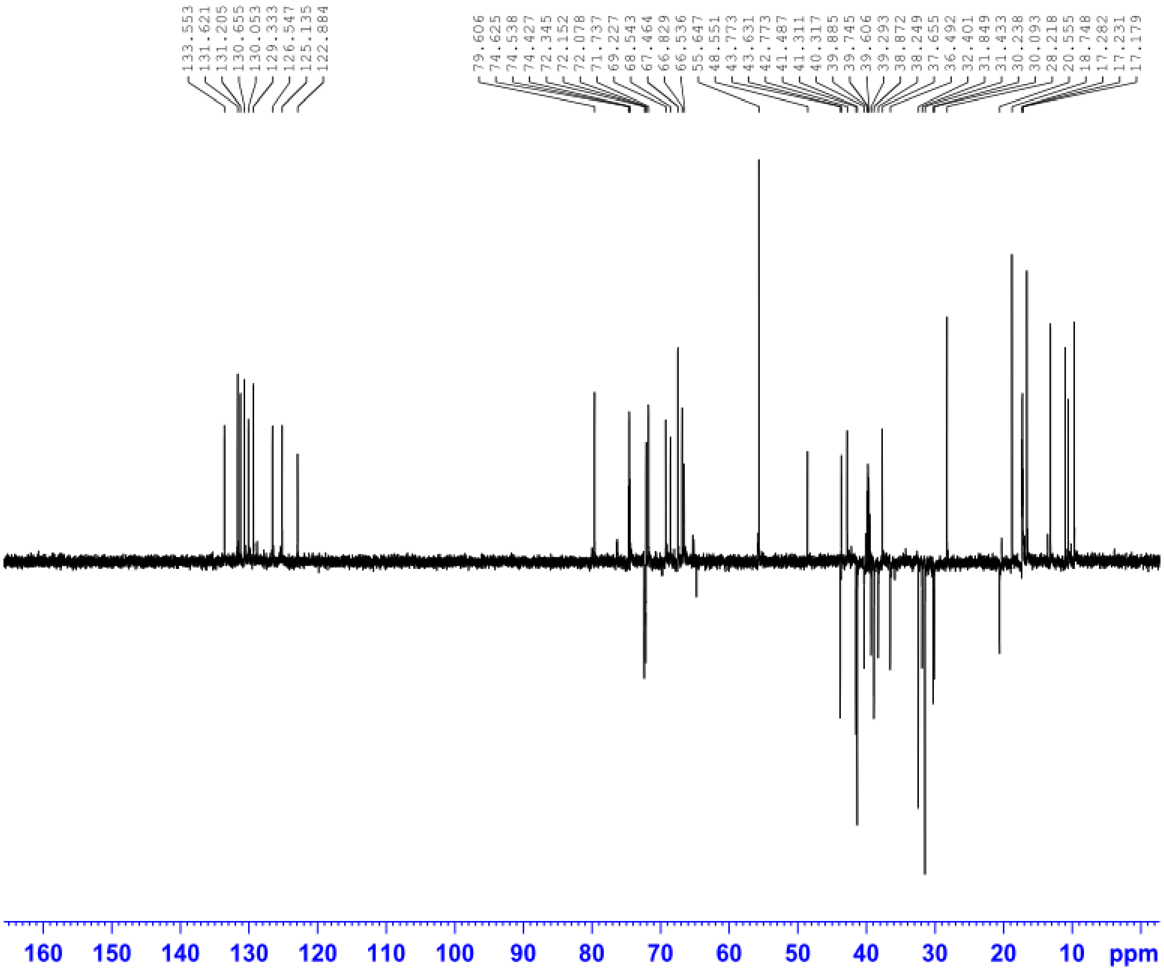

Supplementary Fig. 10. DEPT135 NMR spectrum of lagriene (3).

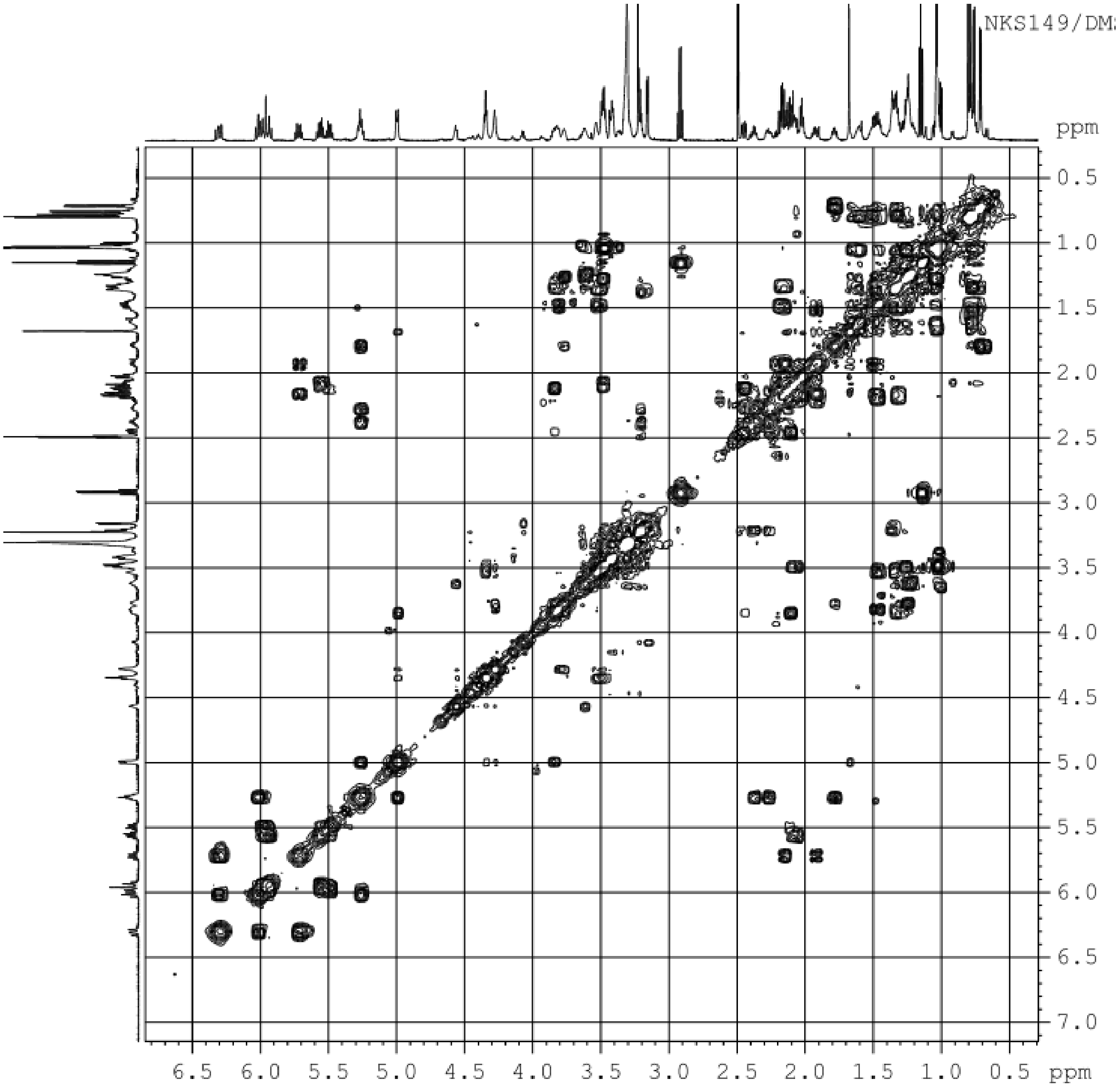

Supplementary Fig. 11. H,H-COSY spectrum of lagriene (3).

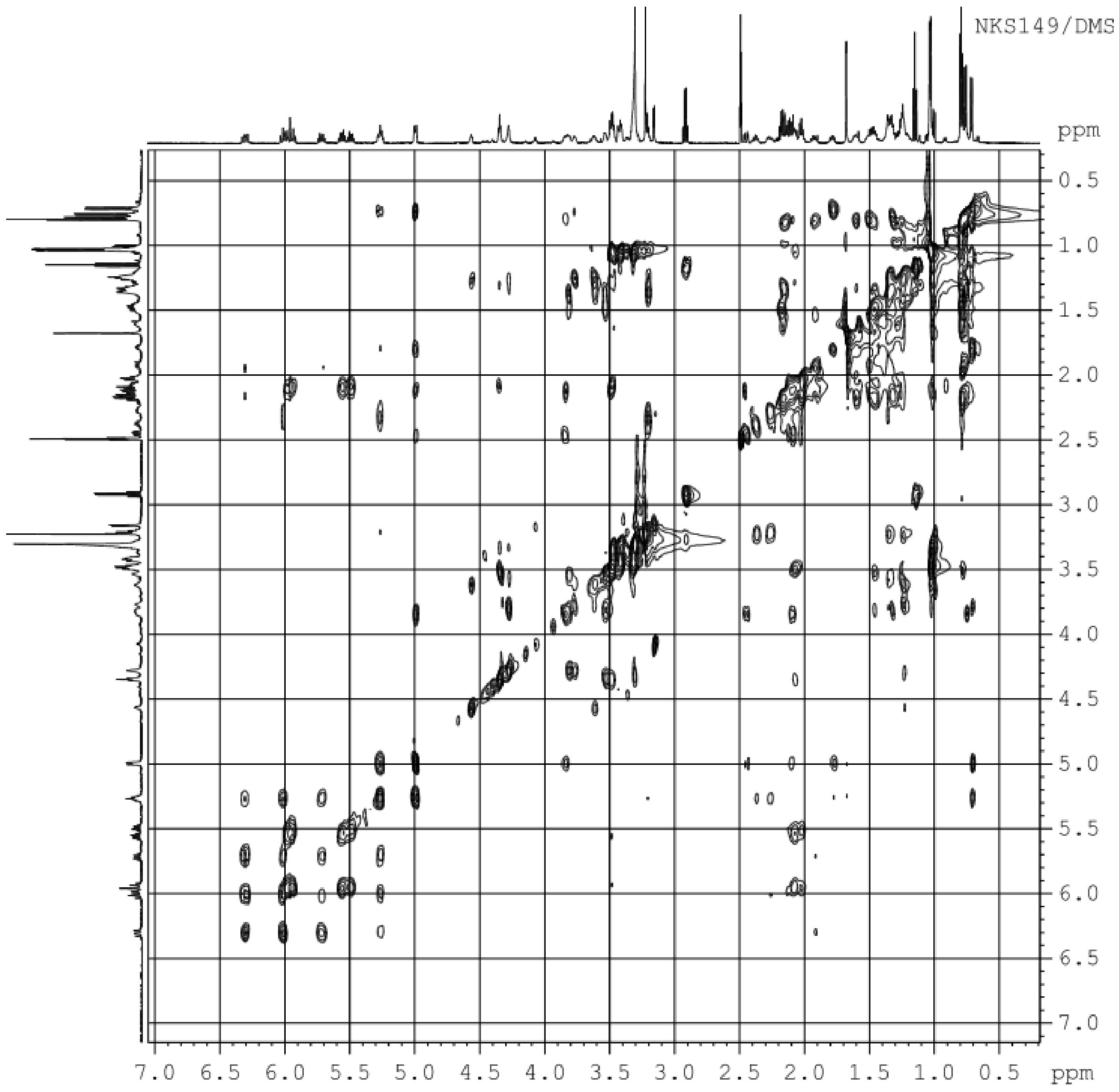

Supplementary Fig. 12. TOCSY spectrum of lagriene (3).

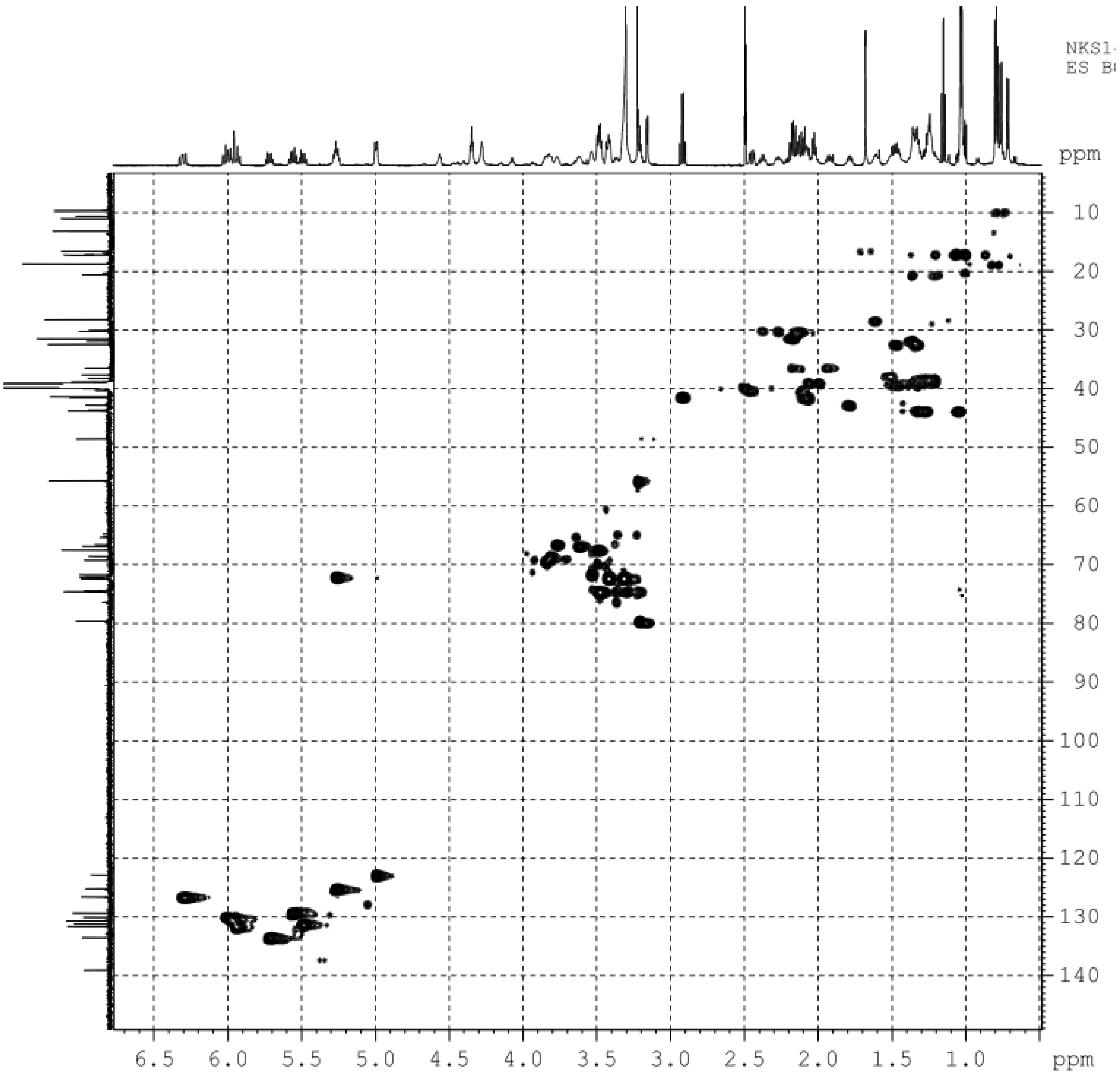

Supplementary Fig. 13. HSQC spectrum of lagriene (**3**).

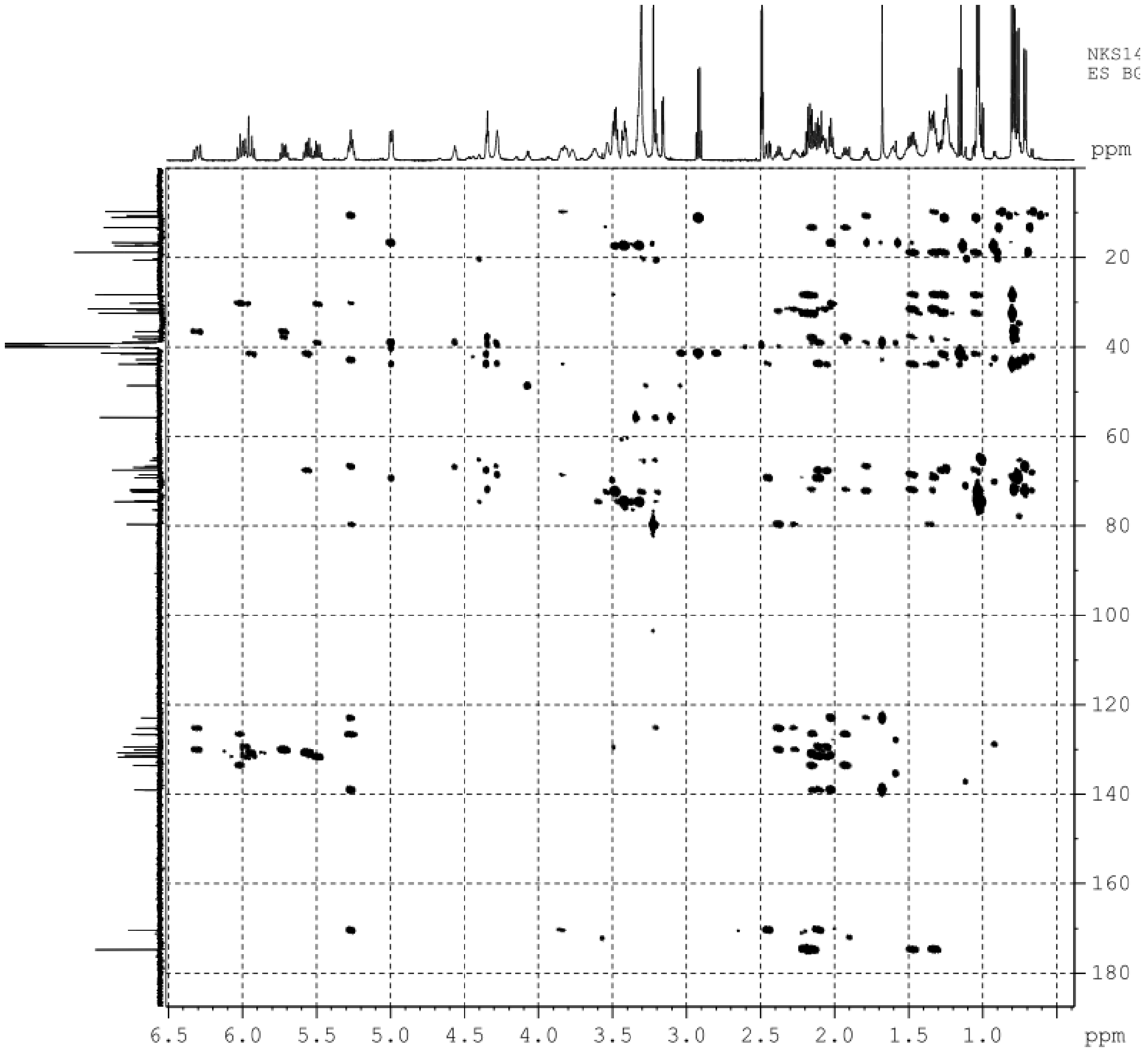

Supplementary Fig. 14. HMBC spectrum of lagriene (3).

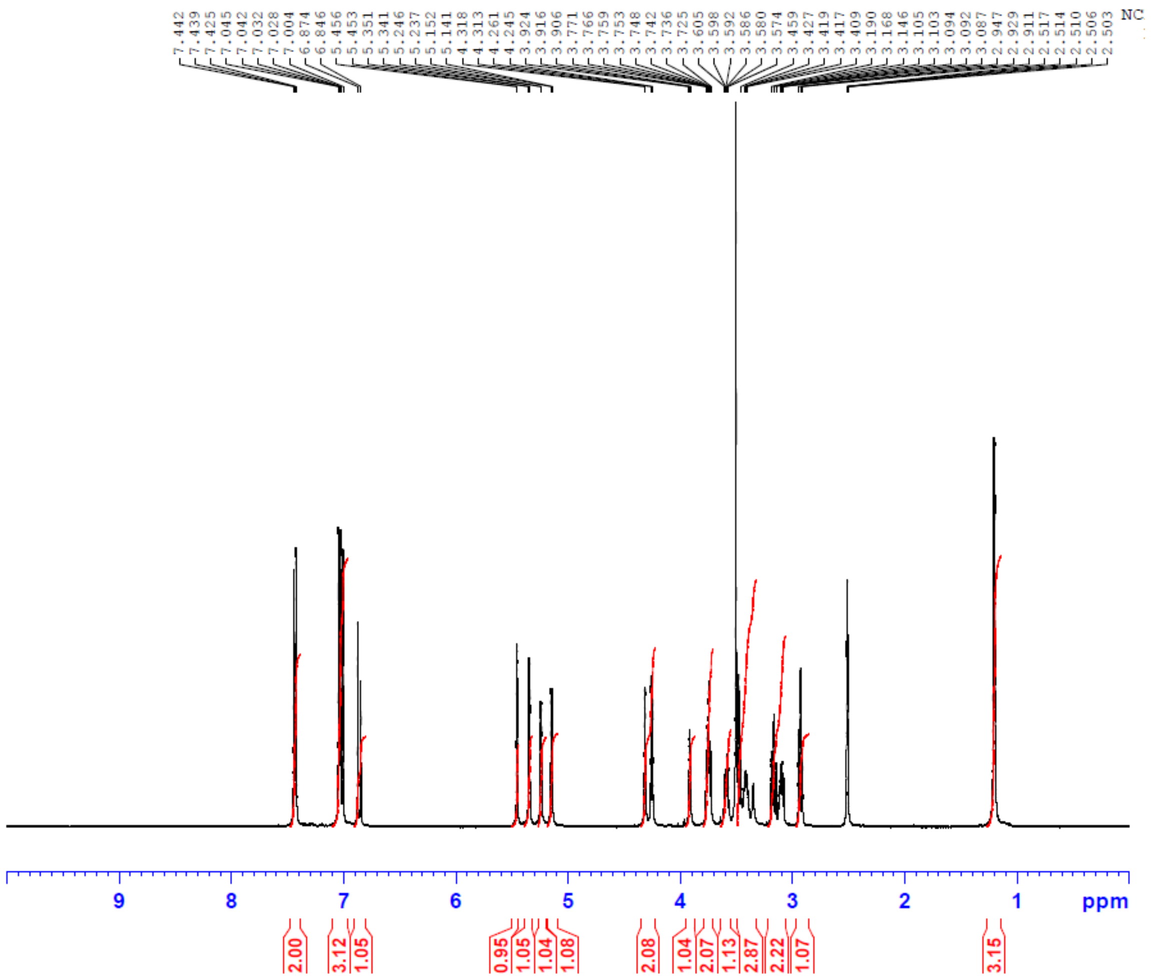

Supplementary Fig. 15. ^{1}H NMR spectrum of sinapigladioside (4).

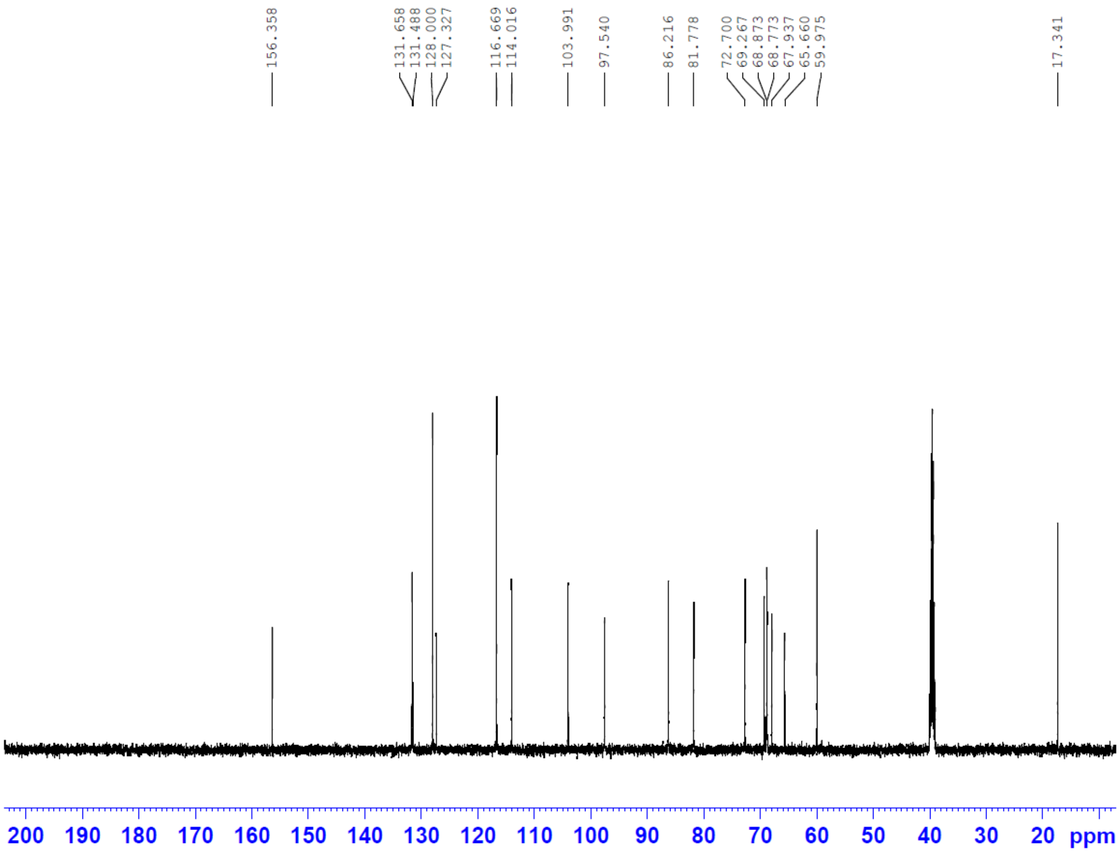

Supplementary Fig. 16. ^{13}**C NMR spectrum of sinapigladioside (4).**

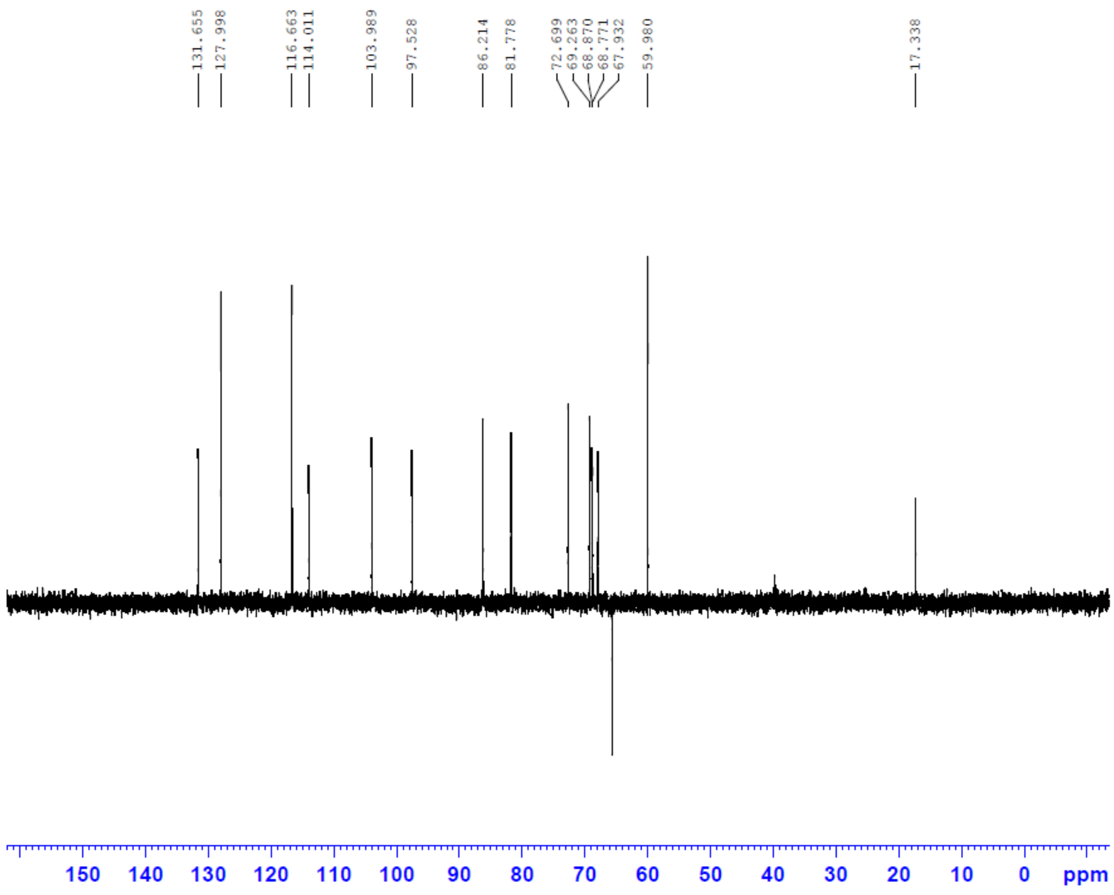

Supplementary Fig. 17. DEPT135 NMR spectrum of sinapigladioside (4).

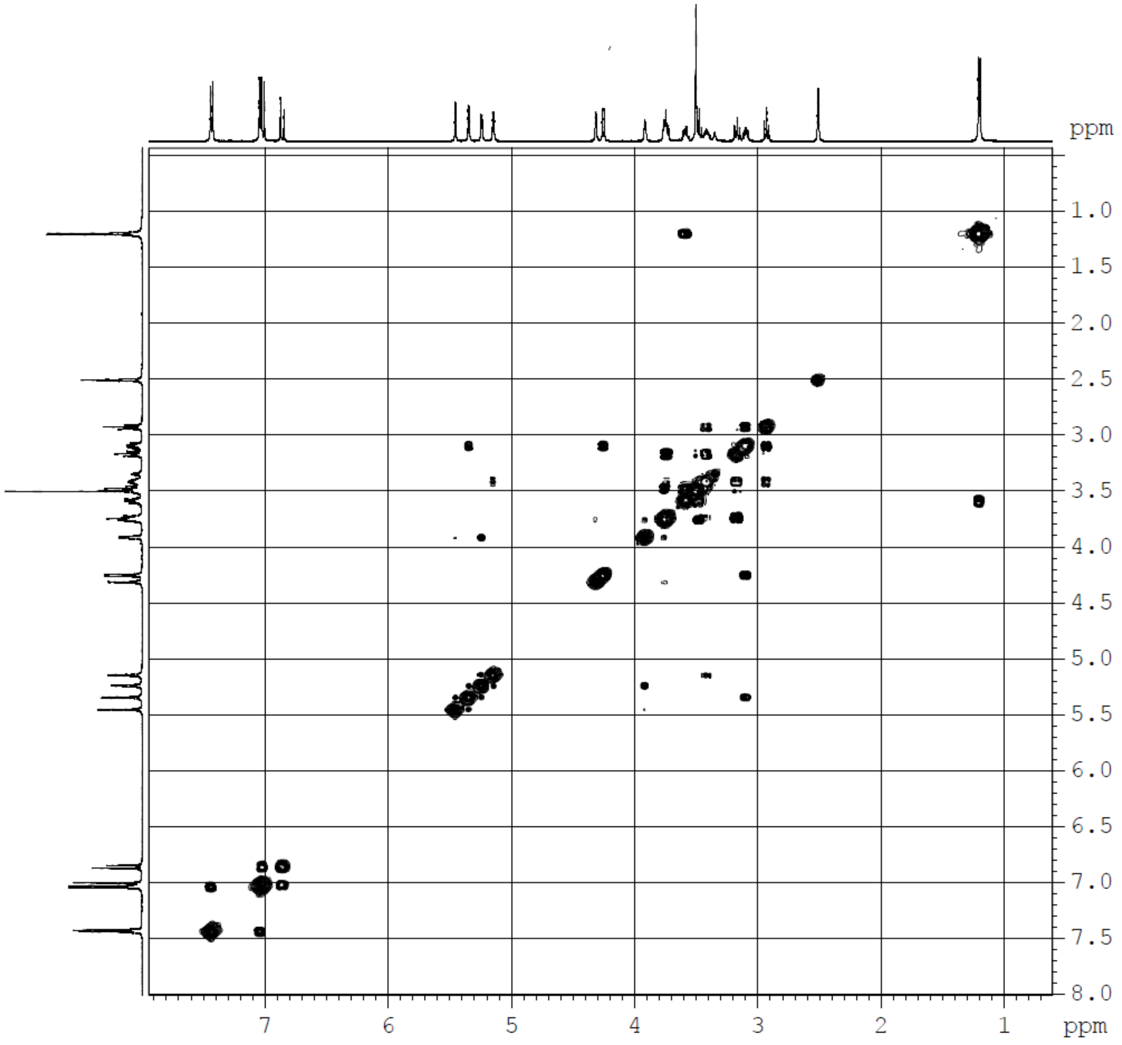

Supplementary Fig. 18. H,H-COSY spectrum of sinapigladioside (4).

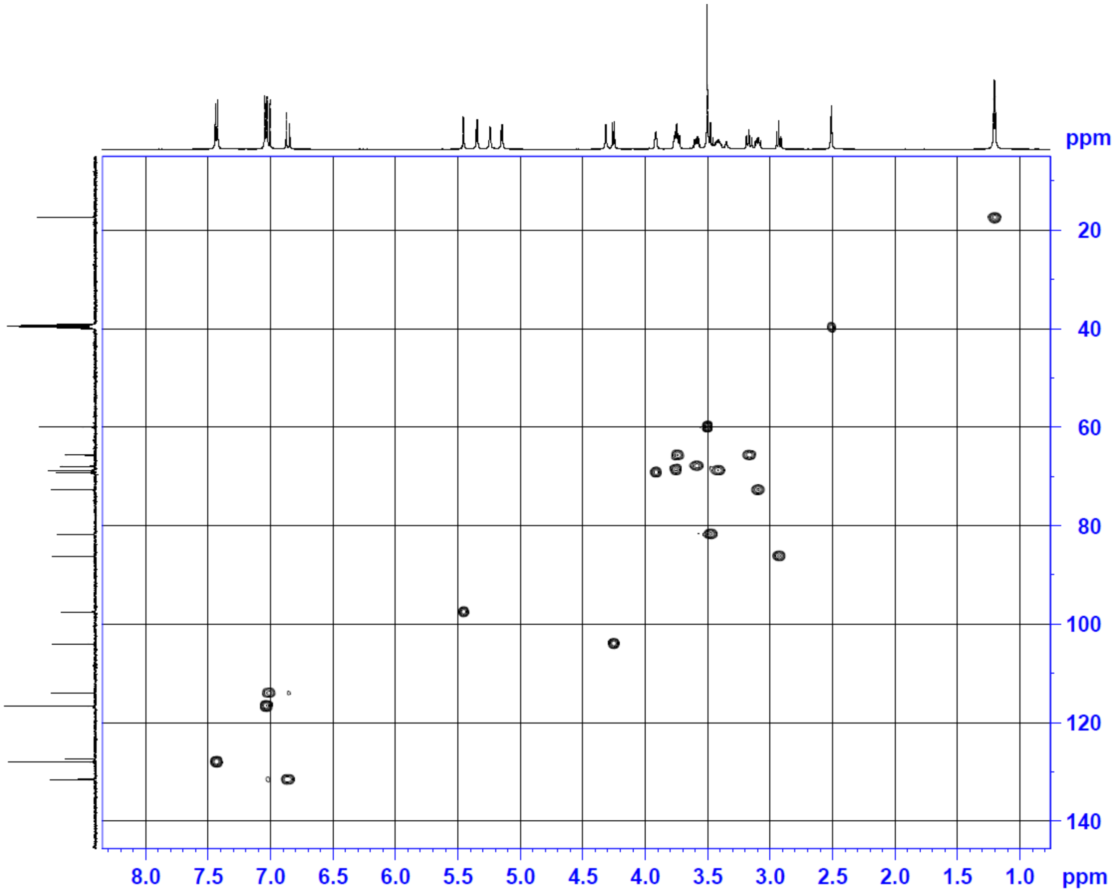

Supplementary Fig. 19. HSQC spectrum of sinapigladioside (4).

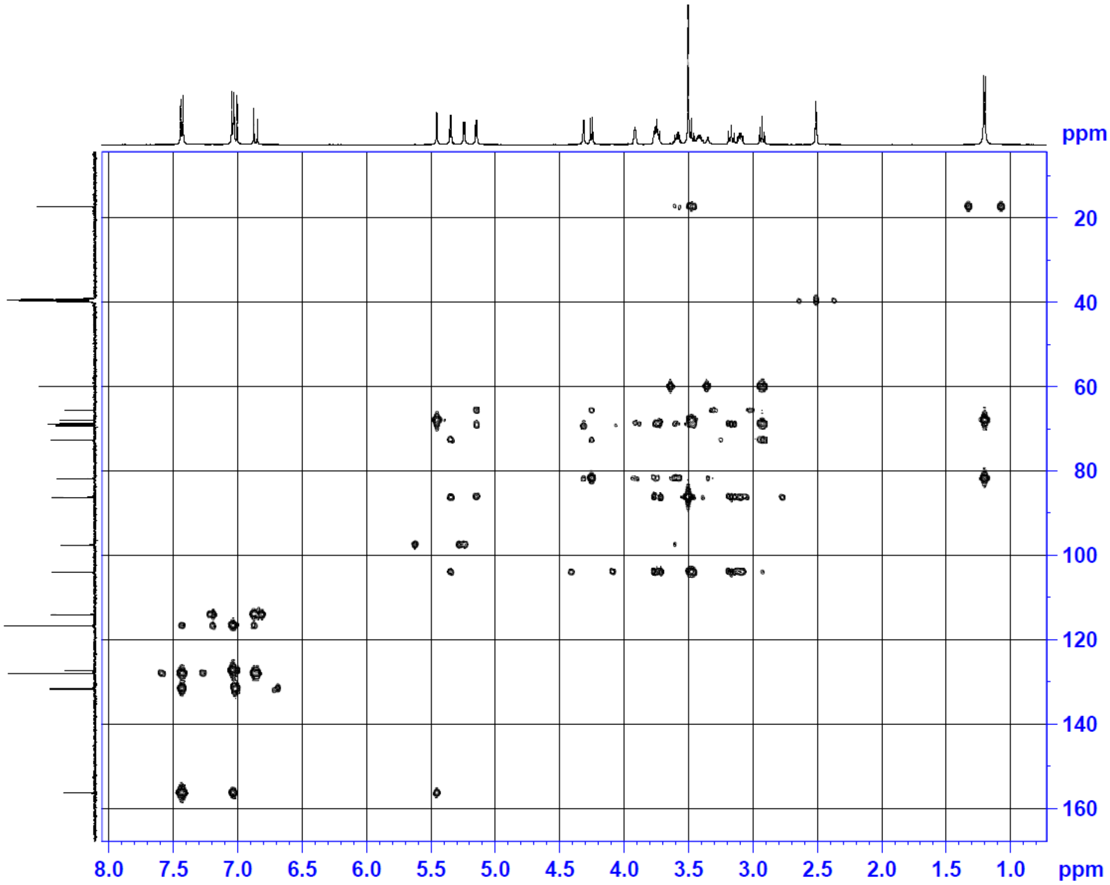

Supplementary Fig. 20. HMBC spectrum of sinapigladioside (4).

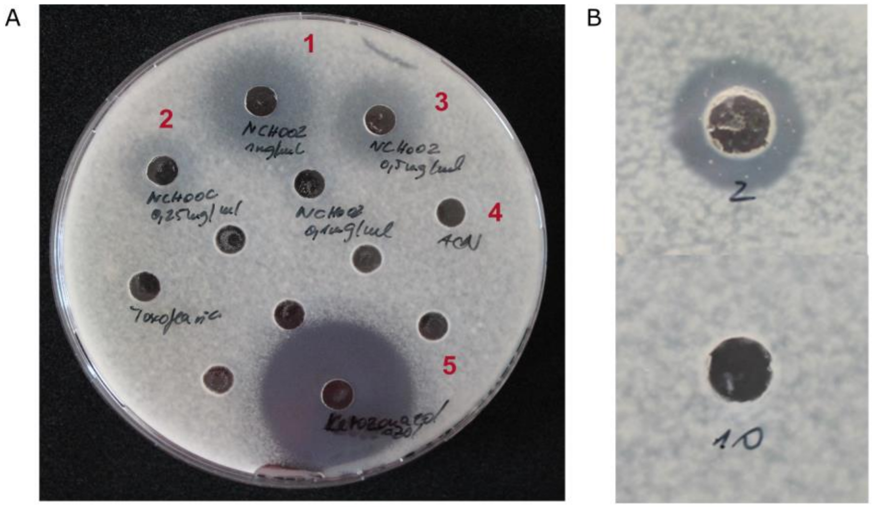

Supplementary Fig. 21. The *B. gladioli* symbionts produce metabolites that are active against *Purpureocillium lilacinum*, a natural fungal antagonist of *L. villosa*. (**A**) Sinapigladioside produced by *L. villosa* StA exhibits antifungal activity against *P. lilacinum.* Wells 1-3 correspond to sinapigladioside at 1, 0.5 and 0.25 mg mL^{-1} respectively, well 4 to the negative control (solvent = acetonitrile) and well 5 to the positive control (Ketoconazol). (**B**) A caryoynencin-containing fraction inhibits the growth of *P. lilacinum in vitro* (upper picture). Negative control (solvent = methanol) (lower picture).

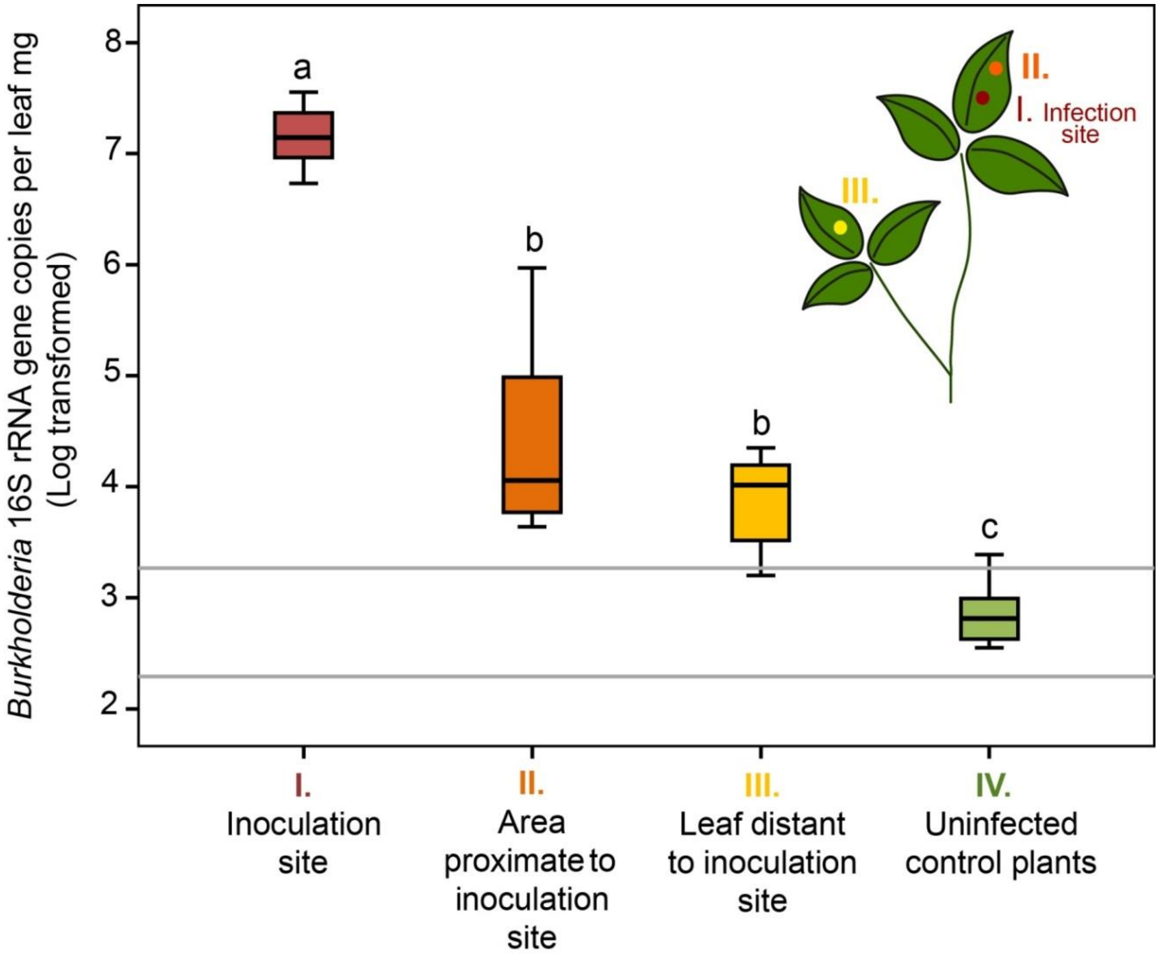

Supplementary Fig. 22. *Burkholderia* symbionts of *L. villosa* can establish a systemic infection in soybean plants. 38 days after infection, *B. gladioli* Lv-StA were found in higher concentration in plant tissues adjacent to the infection site. Significant titers of bacteria were also detected in areas proximate to the inoculation site, as well as in distant leaves (ANOVA, $n = 18$ per group, $p < 0.001$; Tukey test), demonstrating dispersal within the plant, probably via the vascular tissues. Bacteria were quantified using qPCR of a 167 bp region of the 16S rRNA gene as described in the methods section (*Plant fitness effect upon B. gladioli infection*). Gray lines correspond to the maximum and minimum values obtained for negative controls in the same qPCR run. Different letters above boxes represent significant differences according to an ANOVA with Tukey post-hoc tests. The center value of the boxplots represents the median, and the whiskers denote minimum and maximum values.

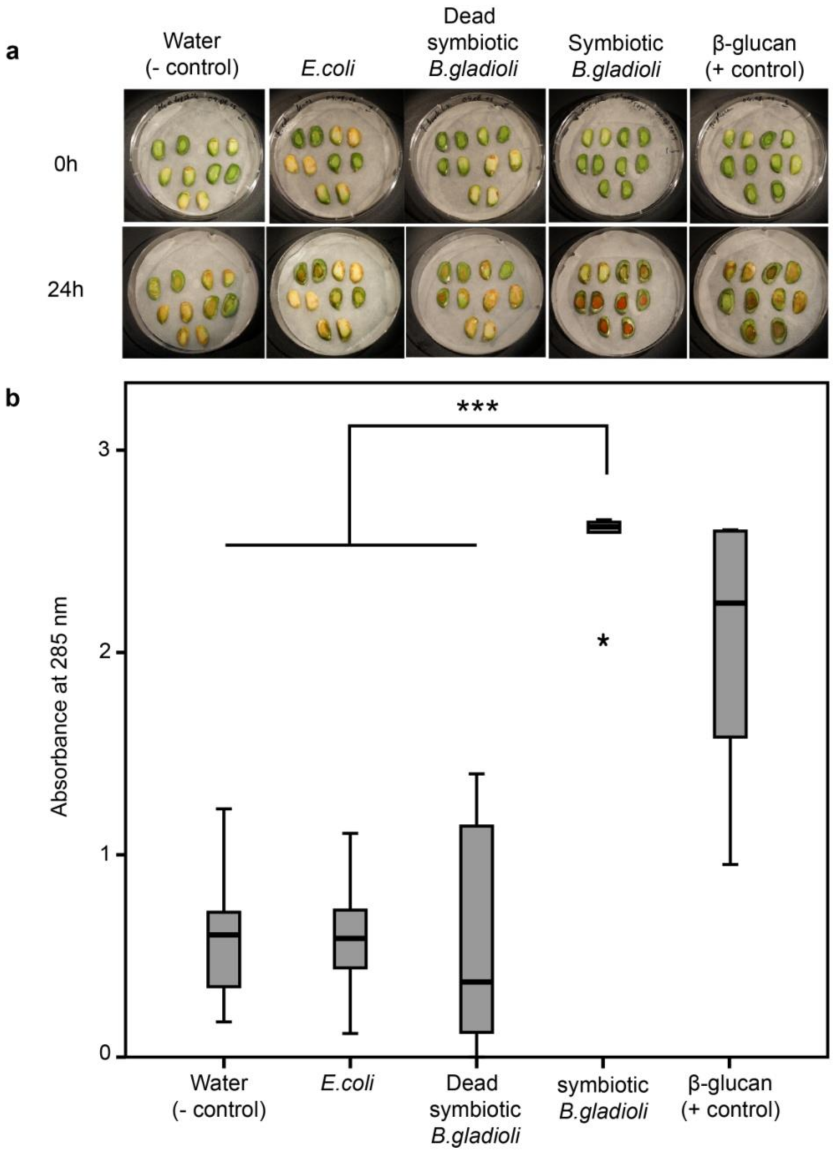

Supplementary Fig. 23. *Burkholderia* symbionts of *L. villosa* elicit a defense response in soybean cotyledons. Cotyledon assay determining glyceollin production by soybean upon inoculation with *B. gladioli* symbionts isolated from *L. villosa*, in comparison with negative (water, *E. coli* and dead *B. gladioli*) and positive controls (β-glucan). (**A**) Red coloration after 24h is indicative of glyceollin production as observed in *B. gladioli* and β-glucan treatments; (**B**) UV-spectrophotometric measurements at 285 nm support significant differences in glyceollin amounts in the different treatments (Kruskall Wallis test with Dunn post-hoc test, $n = 8$ cotyledons per group, ***$p < 0.001$). The center value of the boxplots represents the median, the whiskers denote minimum and maximum values, and the star represents an extreme outlier (more than 3 interquartile ranges from nearest box edge).

Supplementary Table 1. 16S rRNA gene sequences from *Burkholderia* symbionts of Lagriinae, and selected references used for phylogenetic reconstruction (Fig. 2b and Supplementary Fig. 2).

Sequence Id.	*Category*[a]	*Strain*	*Accession*
Lagria nigricollis Symbiont	Lagriinae symbiont	Ln	KT888028
Lagria okinawana Symbiont	Lagriinae symbiont	Lo	KT888029
Lagria rufipennis Symbiont	Lagriinae symbiont	Lr	KT888030
Lagria villosa Symbiont	Lagriinae symbiont	Lv-StA	KT888027
Lagria villosa Symbiont	Lagriinae symbiont	Lv-StB	KU358661
Lagria villosa Symbiont	Lagriinae symbiont	Lv-StC	KU358661
Lagria hirta Symbiont	Lagriinae symbiont	Lh-StG	KT888026
Lagria hirta Symbiont	Lagriinae symbiont	Lh-StH	KU574041
Lagria hirta Symbiont	Lagriinae symbiont	Lh-StM	KU574046
Ecnolagria sp. Symbiont	Lagriinae symbiont	Ec	KT888031
Burkholderia gladioli BSR3 - Rice pathogen	Plant pathogen - *B.gladioli* clade	BSR3	NR_102847
Burkholderia gladioli MS102 - Corn pathogen	Plant pathogen - *B.gladioli* clade	MS 102	EU053154
Burkholderia gladioli CH-2 - Onion pathogen	Plant pathogen - *B.gladioli* clade	CH-2	AY500138
Burkholderia gladioli st3 - Orchid pathogen	Plant pathogen- *B.gladioli* clade	strain 3	DQ090078
Burkholderia gladioli - Rice pathogen	Plant pathogen - *B.gladioli* clade	321gr-6	DQ355169
Burkholderia gladioli pv. *agaricicola*	Fungal pathogen - *B.gladioli* clade	CFBP 3580	GU936678
Burkholderia gladioli pv. *alliicola*	Plant pathogen - *B.gladioli* clade	CFBP 2422	GU936679
Burkholderia gladioli pv. *gladioli*	Plant pathogen - *B.gladioli* clade	CFBP 2427	GU936677
Burkholderia plantarii	Plant pathogen	-	U96933
Burkholderia plantarii - 2396	Plant pathogen	2396	AB183679
Burkholderia plantarii - NBRC104888	Plant pathogen	NBRC104888	AB682222.1
Burkholderia glumae 336gr-1 - Rice pathogen	Plant pathogen	336gr-1	DQ355164.1
Burkholderia glumae - 99gr-4b - Rice pathogen	Plant pathogen	99gr-4b	DQ355167
Burkholderia glumae - PA27.4 - Rice pathogen	Plant pathogen	PA27.4	EF193641.1
Burkholderia caryophylli	Plant pathogen	ATCC 25418	AB021423
Burkholderia endofungorum	Fungal endosymbiont- plant pathogen	HKI 456T	AM420302
Burkholderia rhizoxinica	Fungal endosymbiont- plant pathogen	HKI 454	AJ938142
Burkholderia cepacia	Plant and animal pathogen - BCC	ATCC 25416	U96927
Burkholderia vietnamiensis	Opportunistic animal pathogen - BCC	LMG 10929	AF097534
Burkholderia oklahomensis	Animal pathogen - Pseudomallei group	C6786	DQ108388
Burkholderia fungorum	PBE	LM16225	AF215705
Burkholderia kururiensis	PBE	-	AB024310
Burkholderia nodosa	PBE	Br3437	AY773189
Burkholderia terrae	PBE	KMY02	AB201285
'Ca. Burkholderia kirkii'	PBE	835462	AF475068
Burkholderia sp. [*P.antennata* symbiont]	Insect-associated	PAN136	AB558189
Burkholderia sp. [*P.bicoloripes* symbiont]	Insect-associated	PBI_clone1	AB558203.1
Burkholderia sp. [*R. pedestris* symbiont]	Insect-associated	RPE64	AB558208
Burkholderia sp. [*Tetraponera binghami* gut isolate]	Insect-associated	-	AF459796
Ralstonia pickettii	Outgroup	12J	NC010678

[*]Except for Lagriinae symbionts and insect-associated strains, the categorization is based on previous studies[1,2]

Supplementary Table 2. Toxoflavin (1) (*tox*) biosynthetic genes and predicted functions of proteins.

Gene	Length (bp)	Putative protein	Homologous protein	Accession number	Identity/ Similarity
toxA	612	*S*-Adenosylmethionine-dependent methyltransferase	ToxA (*B. glumae*)	BAA92862.2	98%/98%
toxB	543	GTP cyclohydrolase II	ToxB (*B. glumae*)	BAB88913.1	98%/98%
toxC	1692	WD-repeat protein	ToxC (*B. glumae*)	BAB88914.2	97%/98%
toxD	933	TRP-2	ToxD (*B. glumae*)	BAB88915.1	89%/91%
toxE	1170	Deaminase	ToxE (*B. glumae*)	BAB88916.2	92%/94%
toxR	600	LysR family transcriptional regulator	ToxR (*B.glumae*)	BAC77727.1	88%/90%

Supplementary Table 3. Caryoynencin (2) (*cay*) biosynthetic genes and predicted functions of proteins.

Gene	Length (bp)	Putative protein	Homologous protein	Accession number	Identity/ Similarity
cayA	1557	Fatty acyl-AMP ligase	CayA (*B. caryophylli*)	AIG53814.1	76%/85%
cayB	930	Fatty acid desaturase	CayB (*B. caryophylli*)	AIG53817.1	85%/89%
cayC	975	Fatty acid desaturase	CayC (*B. caryophylli*)	AIG53820.1	85%/91%
cayD	324	Phosphopantetheine attachment site	CayD (*B. caryophylli*)	AIG53823.1	81%/93%
cayE	1101	Fatty acid desaturase	CayE (*B. caryophylli*)	AIG53826.1	88%/94%
cayF	975	Alpha/Beta hydrolase	CayF (*B. caryophylli*)	AIG53829.1	74%/83%
cayG	1197	Cytochrome P450 monooxygenase	CayG (*B. caryophylli*)	AIG53832.1	79%/90%

Supplementary Table 4. Lagriene (3) (*lag*) biosynthetic genes and predicted functions of proteins.

Gene	Length (bp)	Description	Domains
lagD	16284	Polyketide synthase	KS-ACP-ACP ER<br>KS-KR-ACP<br>KS-DH-KR-ACP<br>KS-DH-KR
lagE	11178	Polyketide synthase	ACP KS-KR-ACP<br>KS-ACP-ACP<br>KS-KR
lagF	9927	Polyketide synthase	MT-ACP<br>KS-KR-ACP<br>KS-KR-ACP<br>KS
lagG	14991	Polyketide synthase	DH-KR-ACP<br>KS-KR-ACP<br>KS-MT-ACP<br>KS-KR-ACP<br>KS
lagH	10800	Polyketide synthase	DH-ACP<br>KS-KR-ACP<br>KS-DH-KR-MT-ACP
lagI	17523	Polyketide synthase	ER KS-KR-ACP<br>KS-KR-MT-ACP<br>KS-KR-ACP<br>KS-KR TE

Supplementary Table 5. Proteins encoded upstream and downstream of the *lagD-lagI* gene cluster.

Gene	Length (bp)	Putative protein	Homologous protein	Accession number	Identity/ Similarity
orf-16	1353	MATE efflux protein	sce3194 (*Sorangium cellulosum* Soce56)	CAN93353.1	68%/82%
orf-15	975	Malonyl CoA-acyl carrier protein transacylase	sce3195 (*Sorangium cellulosum* So ce56)	CAN93354.1	47%/63%
orf-14	855	4'-Phosphopantetheinyl transferase	sce5058 (*Sorangium cellulosum* So ce56)	CAN95221.1	41%/56%
orf-13	1173	Malonyl CoA-acyl carrier protein transacylase	Malonyl CoA-acyl carrier protein transacylase (*Burkholderia pseudomallei*)	3G87_A	51%/62%
orf-12	1401	Amidase	sce3176 (*Sorangium cellulosum* So ce56)	CAN93335.1	56%/69%
orf-11	360	Alpha/beta-hydrolase	sce3177 (*Sorangium cellulosum* So ce56)	CAN93336.1	58%/76%
orf-10	3234	Hypothetical protein	SorM (*Sorangium cellulosum* So ce12)	ADN68497.1	38%/51%
orf-9	747	Enoyl-CoA hydratase	sce3179 (*Sorangium cellulosum* So ce56)	CAN93338.1	75%/83%
orf-8	789	Enoyl-CoA hydratase	sce3180 (*Sorangium cellulosum* So ce56)	CAN93339.1	63%/78%
orf-7	1260	3-Hydroxy-3-methylglutaryl CoA synthase	sce3181/EtnO (*Sorangium cellulosum* So ce56)	CAN93340.1	76%/86%
orf-6	1227	Beta-ketoacyl synthase	sce3182/EtnP (*Sorangium cellulosum* So ce56)	CAN93341.1	64%/76%
orf-5	246	Acyl carrier protein	sce3183 (*Sorangium cellulosum* So ce56)	CAN93342.1	68%/81%
orf-4	1395	Malonyl CoA-acyl carrier protein transacylase	sce3184 (*Sorangium cellulosum* So ce56)	CAN93343.1	68%/82%
orf-3	264	Acyl carrier protein	sce3185 (*Sorangium cellulosum* So ce56)	CAN93344.1	60%/81%
orf-2	609	Malonyl CoA-acyl carrier protein transacylase	sce3186/EtnB (*Sorangium cellulosum* So ce56)	CAN93345.1	55%/70%
orf-1	1968	Asparagine synthase	sce3187/EtnC (*Sorangium cellulosum* So ce56)	CAN93346.1	66%/79%
orf+1	486	Hypothetical protein	-	-	-
orf+2	924	Hypothetical protein	-	-	-
orf+3	609	Glutathione-*S*-transferase	Glutathione-*S*-transferase (*Yersinia pestis*)	4G9H_A	47%/65%
orf+4	954	LysR family transcriptional regulator	LysR family transcriptional regulator (*Neisseria meningitidis*)	3HHG_A	34%/54%
orf+5	2124	TonB-dependent siderophore receptor	TonB-dependent siderophore receptor (*Pseudomonas fluorescens*)	3QLB_A	24%/37%
orf+6	858	Molybdate ABC transporter	Molybdate-binding protein (*Xanthomonas axonopodis* pv. *citri*)	3GZG_A	26%/39%
2400/*orf+7*	1092	Oxidoreductase	Luciferase-like monooxygenase (*Bacillus cereus*)	3RAO_A	36%/54%

Supplementary Table 6. Microbial strains used for *in vivo* and *in vitro* activity bioassays.

Species	Strain	Source	Location	Accession Number/ Database Identifier	Database
Purpureocillium lilacinum	LV1	*Lagria villosa* egg	MPI-CE, Jena, Germany	KY630747, KY630748, KY630749	GenBank
Trichoderma harzianum	LESF555	*Achromyrmex heyeri* fungal garden	Sentinela do Sul, Rio Grande do Sul, Brazil	KT279018, KT278950, KT278904	GenBank
Beauveria bassiana	LESF477	*Atta* sp. female alate	Botucatu, Sao Paulo, Brazil	CRM 1216	CRM - UNESP
Candida albicans	BMSY 212	Jena Microbial Resource Collection	Jena, Germany	STI50163	JMRC - Friedrich Schiller University
Penicillium notatum	JP 36	Jena Microbial Resource Collection	Jena, Germany	SF011781	JMRC - Friedrich Schiller University
Aspergillus fumigatus	ATCC46645	Jena Microbial Resource Collection	Jena, Germany	STH00450	JMRC - Friedrich Schiller University
Sporobolomyces salmonicolor	SBUG 549	Jena Microbial Resource Collection	Jena, Germany	ST035974	JMRC - Friedrich Schiller University
Bacillus thuringiensis	DSM2048	Jena Microbial Resource Collection	Jena, Germany	STI11409	JMRC - Friedrich Schiller University
Bacillus subtilis	ATCC 6633	Jena Microbial Resource Collection	Jena, Germany	STI10880	JMRC - Friedrich Schiller University
Brevibacillus laterosporus	ATCC 31932	Jena Microbial Resource Collection	Jena, Germany	STI11381	JMRC - Friedrich Schiller University
Staphylococcus aureus	SG 511	Jena Microbial Resource Collection	Jena, Germany	STI10760	JMRC - Friedrich Schiller University
Escherichia coli	SG 458	Jena Microbial Resource Collection	Jena, Germany	ST033699	JMRC - Friedrich Schiller University
Pseudomonas aeruginosa	SG 137	Jena Microbial Resource Collection	Jena, Germany	ST033772	JMRC - Friedrich Schiller University
Pseudomonas aeruginosa	K 799/61	Jena Microbial Resource Collection	Jena, Germany	ST033771	JMRC - Friedrich Schiller University
Staphylococcus aureus	134/93 (MRSA)	Jena Microbial Resource Collection	Jena, Germany	STH00435	JMRC - Friedrich Schiller University
Enterococcus faecalis	1528 (VRE)	Jena Microbial Resource Collection	Jena, Germany	ST033700	JMRC - Friedrich Schiller University
Mycobacterium vaccae	IMET 10670	Jena Microbial Resource Collection	Jena, Germany	STI10670	JMRC - Friedrich Schiller University

Supplementary References

1. Z. R. Suárez-Moreno *et al.*, Common features of environmental and potentially beneficial plant-associated *Burkholderia*. *Microb. Ecol.* **63**, 249–266 (2012).

2. B. Verstraete, S. Janssens, E. Smets, S. Dessein, Symbiotic ß-proteobacteria beyond legumes: *Burkholderia* in Rubiaceae. *PLoS One*. **8** (2013), doi:10.1371/journal.pone.0055260.
